# An Overview: Highly Efficient Inhibitors of Carbon Steel

**DOI:** 10.3390/molecules30193994

**Published:** 2025-10-05

**Authors:** Giselle Gómez-Sánchez, Víctor Díaz-Jiménez, Paulina Arellanes-Lozada, Irina V. Lijanova, Janette Arriola-Morales, Maribel Castillo-Morales, Natalya Victorovna Likhanova, Octavio Olivares-Xometl

**Affiliations:** 1 Facultad de Ingeniería Química, Benemérita Universidad Autónoma de Puebla, Ciudad Universitaria, Av. San Claudio y 18 Sur, Col. San Manuel, Puebla 72570, Mexico; giselle.gomezs@alumno.buap.mx (G.G.-S.); janette.arriola@correo.buap.mx (J.A.-M.); maribel.castillo@correo.buap.mx (M.C.-M.); 2 Departamento de Ingeniería en Biotecnología, Universidad Politécnica Metropolitana de Puebla, Popocatépetl S/N, Reserva Territorial Atlixcáyotl, Tres Cerritos, Puebla 72480, Mexico; vmdjdj@gmail.com; 3 Instituto Mexicano del Petróleo, Dirección de Investigación, Eje Central Norte Lázaro Cárdenas No. 152, Col. San Bartolo Atepehuacan, G. A. Madero, Mexico City 07730, Mexico; parellanes@imp.mx; 4 Instituto Politécnico Nacional, CIITEC, Cerrada Cecati S/N, Col. Santa Catarina de Azcapotzalco, Mexico City 02250, Mexico; irinalijanova@yahoo.com.mx

**Keywords:** carbon steel, adsorption, corrosion inhibitor, acid corrosion, electrochemical techniques

## Abstract

The synthesis of new corrosion inhibitors (CIs) has been significantly encouraged in recent years because of the important economic losses that the corrosion of steel alloys exposed to corrosive aqueous media cause. In the oil industry, a common and unavoidable practice is the use of CIs whose chemical configuration has to be conceived in such a way that these compounds can withstand specific operation conditions at low concentrations (parts per million). Due to the fact that current information on CIs is very vast, the present review aimed to narrow it down by analyzing the contributions to the field of corrosion control published in the last five years featuring CIs with inhibition efficiency (IE) ≥ 90% at concentrations below 100 ppm in HCl, H_2_SO_4_ and H_2_S media and mainly evaluated by weight loss and electrochemical techniques.

## 1. Introduction

Steel and its alloys have been essential for decades, playing a major role in numerous industrial sectors. Their high resistance, durability and adaptability have set them as indispensable materials that have been used extensively and massively in diverse industrial fields ranging from high technology devices and machinery to big scale industrial production processes. Furthermore, their complete recyclability and global accessibility make them sustainable and fundamental for modern industrial development [1]. The vulnerability of steel to corrosion in industrial environments is a significant challenge to deal with. When exposed to humidity, salts, chemical products or under the extreme conditions of chemical processes such as high temperature, pressure and flow, steel goes through gradual decay that modifies its original physicochemical properties and shortens its useful life [2].

**Figure 1:**
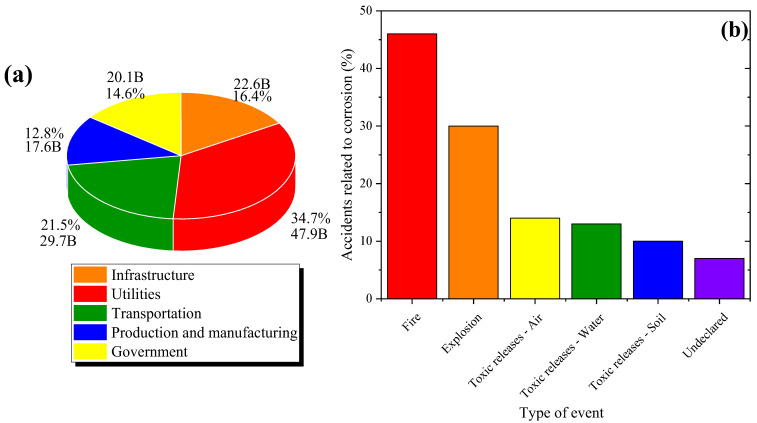
Statistics on the general implications of corrosion: (**a**) corrosion percentage affecting major sectors in the USA in 2014, and direct corrosion costs in these sectors [8], and (**b**) percentage of refinery accidents related to inadequate corrosion control. Information extracted by a European Union report in 2012 (note that there may be more than one type of event per accident) [9].

Corrosion damage represents a critical problem at the industrial level, and its study and control have important implications that can be classified into three categories [3,4,5,6]:

- **Economy:** The economic impact of corrosion is really high and considerably affects the economy of multiple countries. As for costs, most countries devote approximately between 2 and 5% of the national Gross Domestic Product (GDP) to mitigate the effects of this phenomenon [7]. It includes direct and indirect costs associated with corrosion, from reparation and maintenance expenses to losses caused by production interruptions and competitivity diminution. The annual direct cost of corrosion damage suffered by five major sectors has been estimated at USD 137.9 billion, equivalent to USD 276 billion in the total US economy, with utilities and transportation sectors being the most affected, as shown in Figure 1a [8].

From the perspective of a country’s economy, it is vital that effective strategies aimed at minimizing losses caused by corrosion be adopted. With the increasing technological and industrial advancements, the use of metals and special alloys has grown quickly, where any action succeeding in preventing corrosion efficiently represents significant benefits [6].

- **Security:** Corrosion can compromise the structural integrity of equipment and infrastructure, increasing the risk of grave accidents; its suitable control protects both the individual and population, and improves general operative security. A European Union report studied 99 significant refinery accidents related to corrosion in the USA and OECD countries from 1965 to 2012. As shown in Figure 1b, 76% of these studied accidents involved potentially high consequences such as fire or explosions [9].
- **Preservation of materials and metallic resources:** To reduce the loss of finite resources, inhibitors and resistant alloys have been employed; thus, extending the useful life of materials and diminishing extraction, which reduces the demand for the production of new alloys and the environmental impact associated with mineral extraction and refining [10].

Corrosion inhibition is an important and widely used strategy for the long-term protection of carbon steels [1], whose selection depends on its economic feasibility, capacity to protect the base metal and minimal negative environmental impact [11]. In this context, the Oslo-Paris (OSPAR) Commission and the Registration, Authorization, and Restriction of Chemicals (REACH), belonging to the European Community, proposed new requisites for the use of chemical substances in order to encourage the research and development of alternative and ecological inhibitors that keep complying with their purpose at an industrial level [12]. Corrosion inhibitors (CIs) stand out because of their easy-to-use features, low cost and efficiency for inhibiting corrosion significantly with minimal dosage, in general, in parts per million (ppm) [13]. The addition of organic compounds as CIs has become an effective alternative for retarding the corrosion process of steel, especially in some industrial processes for the production of oil and gas, where acid corrosive media are used or susceptible to be formed [14]. The advances in the development of new CIs have allowed the availability of feasible alternatives to replace inorganic CIs because these compounds not only exert negative environmental effects through bioaccumulation and difficult biodegradability but also affect human health due to their toxicity and carcinogenic features. Although it has been stated that there are strategies for the right use of inorganic CIs to reduce their impact on the environment and humans, they are still obsolete. According to the foregoing, in the current literature, a more viable alternative to inorganic CIs is the use of green CIs, where many organic compounds and plant extracts represent excellent options.

The corrosion inhibition mechanism starts from the competition and replacement (diffusion processes) of molecules/ions from the acid environment by CI molecules on active sites of the steel surface through the metal–solution interface with their further adsorption (physical and/or chemical) through four possible interaction types: (*i*) electrostatic attraction between charged molecules and the charged metal, (*ii*) interaction between unshared electron pairs in a molecule and the metal, (*iii*) interaction between *p* electrons and the metal and (*iv*) combinations of the previous interactions, where the inhibitor behavior can be described by physical adsorption, chemisorption or complexation with the metal surface [15].

The study field of CIs emphasizes the relationship between their adsorption process and chemical structure, focusing on the understanding of structural and electronic properties that include functional groups, the presence of electron-repulsing or electron-donating groups, electron density of the donor atoms, donor character of *p* orbitals, steric factors and aromaticity, among others. Likewise, it is important to know the charge, nature and electronic characteristics of the metal surface, as well as the adsorption process of the acid corrosive environment (mostly water and other ionic species), electrochemical potential in the metal–solution interface and other relevant properties of the system such as temperature, flow rate, and pH, among others [16]. It is well known that inhibition efficiency (IE) and, as a consequence, the degree of metal surface covered by a protecting film consisting of an organic inhibitor are significantly related to its functional groups and structures such as unsaturated bonds, *π* bonds, aromatic rings, heteroatoms (like O, N. S, P), etc., which provide characteristics such as high density electron clouds and polarity that enable their spontaneous adsorption on the steel surface [17].

Understanding the mechanism through which organic CIs interact with metal surfaces provides the basis to explore their application in aggressive media such as hydrochloric, sulfuric and hydrosulfuric acids, among others. In addition, volumetric flow, temperature, pressure, pH, etc., represent critical variables that affect CI performance by drastically diminishing the IE. The following sections analyze CIs that were reported on in recent years and that achieved IEs above 90% in different corrosive media.

## 2. Highly Efficient Inhibitors of Carbon Steel in Acidic Environments

### 2.1. Corrosion Inhibition of Carbon Steel by Hydrochloric Acid

Hydrochloric acid (HCl), widely present in industrial applications such as chemical synthesis and the cleaning of heavy metals, represents one of the most common and challenging corrosive media. Its frequent use is one of the main reasons for the high indices of corrosion damage both under controlled and uncontrolled conditions. Industrial sectors such as metallurgical, oil and gas extraction, water treatment, chemical production and construction face significant corrosion risks because of either the controlled use or accidental formation of hydrochloric acid [18,19,20]. The intentional utilization of HCl in these processes is more economical, efficient and easy to handle in comparison to other mineral acids [21]. Notwithstanding, its presence is a common corrosion cause in crude oil distillation units; at temperatures above 393 K (120 °C), hydrogen chloride (HCl) can be generated by the decomposition of sodium chloride (NaCl), calcium chloride (CaCl_2_) and magnesium chloride (MgCl_2_) salts. Under these conditions, water vapor at the superior system condensates and these water molecules in liquid state react with hydrogen chloride to produce hydrochloric acid [22,23]. In addition, water can absorb ammonia, which by reacting with hydrogen chloride, forms ammonium chloride (NH_4_Cl). These compounds are highly acidic and can provoke localized corrosion like pitting corrosion [24,25]. Especially in the oil industry, the high corrosivity of HCl generates grave damage in steel pipelines. This corrosive agent is responsible for the occurrence of leaks, material failure, loss of structural integrity and flow perturbations, which significantly increase production costs due to cleaning tasks and environmental remediation [26].

The corrosion process of steel by HCl can be described by Reaction (1), which is divided into two essential partial electrochemical reactions: anodic (Reactions (2)–(7)) and cathodic (Reactions (8)–(10)). At first, Fe, the main component of steel, is oxidized and releases Fe^2+^ ions to the electrolyte, generating free electrons circulating through the metal. In this mechanism, the adsorbed intermediates [FeOH]_ads_ and [FeClOH]_ads_ (Reactions (2) and (6)) play a major role in establishing the steel dissolution rate. Furthermore, the presence of Cl^−^ ions from the corrosive environment can promote the intermediate [FeOH]_ads_ dissolution in acid media [27].

*General reaction:*

(1) $$Fe_{\left(s\right)}+2H^{+}\to Fe^{2+}+H_{2(g)}$$

*Anodic reaction in acid aqueous solution:*

(2) $$Fe+H_{2}O↔{\left[FeOH\right]}_{ads}+H^{+}+e^{-}$$

(3) $${\left[FeOH\right]}_{ads}↔{\left[FeOH\right]}_{ads}^{+}+e^{-}$$

(4) $${\left[FeOH\right]}_{ads}^{+}+H^{+}↔Fe^{2+}+H_{2}O$$

*Anodic reaction in acid aqueous solution and presence of Cl^−^ ions:*

(5) $$Fe+H_{2}O+Cl^{-}↔{\left[FeClOH\right]}_{ads}^{-}+H^{+}+e^{-}$$

(6) $${\left[FeClOH\right]}_{ads}^{-}↔{\left[FeClOH\right]}_{ads}+e^{-}$$

(7) $$\left[FeClOH\right]+H^{+}↔Fe^{2+}+Cl^{-}+H_{2}O$$

*Cathodic reaction in acid solution:*

(8) $$Fe+H_{3}O^{+}⥂Fe{\left(H_{3}O^{+}\right)}_{ads}$$

(9) $$Fe{\left(H_{3}O^{+}\right)}_{ads}+e^{-}\to Fe{\left(H_{3}O\right)}_{ads}$$

(10) $$Fe{\left(H_{3}O\right)}_{ads}+H_{3}O^{+}+e^{-}\to Fe+H_{2}↑+H_{2}O$$

To mitigate such effects, CIs capable of reducing the metal dissolution rate and extending its useful life are employed. Their evaluation is carried out by means of electrochemical techniques such as Tafel polarization and electrochemical impedance spectroscopy (EIS).

Table 1 shows numerous and diverse CIs that have reached IE ≥ 90% at concentrations below 100 ppm; this list features from functionalized cyclic organic compounds to polymers and ionic liquids (ILs) for different types of carbon steel in HCl corrosive media.

Diterpenoids represent a diverse group of chemical compounds, characterized by a 20-carbon atom skeleton. Depending on their main structure, these compounds are classified into different categories such as acyclic, monocyclic, bicyclic, tricyclic, tetracyclic and macrocyclic diterpenes. These compounds usually present polyoxygenated forms that include hydroxyl and keto (C=O) groups, which are frequently esterified with aliphatic or small aromatic acids [54]. In this context, Ettefagh Far et al. [28] studied dehydroabietylamine (DHAA), a diterpenoid compound consisting of connected single and aromatic rings, as well as an amine group, in the protection of mild steel in 1.0 M HCl. This compound reached an IE of 91% at the highest evaluated concentration (25 ppm), which was attributed mainly to its adsorption through the N atom that can donate lone electron pairs to the metal surface and block H^+^ discharge.

Other organic compounds with heteroatoms were studied as possible CIs, which were based on pyrimine [29] and aniline [30,31]. For the first study, El Ouadi et al. [29] employed the structure 1,5-dimethyl-1H-pyrazolo[3,4-d]pyrimidin-4(5H)-one (MF3), which reached IEs of 90.5 and 93% at concentrations of 1 × 10^−4^ and 1 × 10^−3^ M, respectively; such results were associated with the adsorption of the protonated forms of N atoms, which facilitated adsorption, forming an insoluble and stable film on the steel surface. Additionally, these researchers suggested that the carbonyl group (C=O) also contributed to the inhibition process because of having unshared electrons in the O atom, which along with N, can interact with empty Fe orbitals on the surface. By means of DFT analysis, it was found that the whole molecule was capable of donating and receiving electrons in the ring and N/O heteroatom regions, mainly.

In contrast, aniline-based compounds worked differently, as shown by Alamiery et al. [30], who added an oxazole group. These authors stated that oxazoles feature multiple advantages in comparison to other groups such as their ecological aspect, profitability, synthesis easiness and high IEs of 92.8 and 94.7% at 0.4 and 0.5 mM, respectively. The DFT analysis suggested that the highest contribution came from aniline in 3-OYA because the highest–occupied molecular orbital (HOMO) and lowest–unoccupied molecular orbital (LUMO) were located mainly over this molecular region. In addition, the authors suggested an inhibition mechanism, where the interaction between the molecule and Cl^−^ ions, which charged the metal surface negatively, and the possible attraction and adsorption of the protonated forms on these sites are emphasized. The formation of coordination bonds with unshared (heteroatoms; physisorption) and/or *π* (aromatic ring; chemisorption) electrons was also suggested. Meanwhile, Shainy et al. [31] evaluated a dianiline group bound through sulfane groups. In this study, a 2-amino thiophenol dimer containing N, S and an aromatic ring in its structure, 2,2^1^ disulfane-diyl-dianiline (DDD), was employed, achieving IEs of 90.1 and 94.0% at 40 and 50 ppm in 0.5 M HCl. The authors suggested that DDD was adsorbed through *π* electrons of both aromatic rings and unbound electrons of S atoms in the molecule, generating coordinate bonds with Fe. In addition, the effect of temperature on the inhibition process was studied and a slight increase in efficiency at 303 K was observed, with a further diminution by increasing the temperature again; such a behavior pattern can be associated with the steric effect between aromatic rings in the surrounding molecules.

Other nitro–sulfur-based compounds were studied by Huong et al. [32], Ahmed et al. [18] and El-hajjaji et al. [33]. In the first work, the authors found that N–phenylthiourea achieved IEs between 90.5 and 94.9% at concentrations from 1 × 10^−4^ to 5 × 10^−3^ M. This behavior was associated with the molecule planar nature and the N and S atoms, which can be easily adsorbed on steel. The protonation of N-phenylthiourea was suggested, which increased its dissolution and allowed its attraction toward Cl^−^ ions, which were previously adsorbed on the metal surface and worked as adsorption bridges. It was also proposed that the molecule could be adsorbed partially on the metal surface through chemisorption, implying the formation of coordinate bonds with the aromatic ring and unprotonated–lone electrons from the S and/or N atoms. By DFT analysis, it was found that the molecule HOMO and LUMO were located mainly on the S atom and surrounding N atoms. In the second study [18], 1-mesitylethanone thiosemicarbazone (MTSC) was analyzed as a possible CI, which achieved an IE of 92.3% at 0.4 M in 1.0 M HCl solution, where the formation of a physical barrier limited the attack of Cl^−^ ions on the metal surface. In addition, the authors related the inhibiting properties of MTSC to molecular stiffness. However, such properties were affected by the temperature, thus diminishing its effectiveness as a CI. Like the previous case, the DFT analysis showed the distribution of the HOMO and LUMO lobes mainly over the region where the N and S heteroatoms were located. In the corrosion inhibition mechanism, it was suggested that the N and S atoms of the thiosemicarbazone group formed coordinate bonds, whereas through the aromatic ring, just a simple physisorption process occurred. Finally, in the third study [33], the Molecular Dynamic (MD) analysis of the molecule 1-Octyl-2-(octylthio)-1H-benzimidazole (T3) in an Fe (111) matrix was featured, achieving IEs of 90.4 and 92.3% at 1 × 10^−4^ and 1 × 10^−3^ M, respectively; the authors indicated that the main adsorption centers were nitrogen, sulfur and the system of *π* electrons.

On the other hand, Ouakki et al. [34] developed work based on structures derived from quinazolinone. These nitrogenated heterocycle compounds consist of a benzene ring linked to a pyrimidine ring [55]. The two evaluated compounds, 3,3-dimethyl-12-(4-nitrophenyl)-3,4,5,12-tetrahydrobenzo[4,5]imidazo[2,1-b]quinazolin-1(2 H)-one [Q-NO_2_] and 12-(4-methoxyphenyl)-3,3-dimethyl-3,4,5,12-tetrahydrobenzo[4,5]imidazo[2,1-b]quinazolin-1(2H)-one [Q-–OMe], achieved IEs of 93.5 and 91.2% at 1 × 10^−4^ M and 1 × 10^−6^ M, respectively. Notwithstanding, the IE decreased with the increasing temperature. The analysis by SEM/EDS and FT-IR supported the hypothesis of the CI being adsorbed on the steel surface by locating peaks associated with C and O. The authors suggested that the studied molecules were protonated due to the medium pH; the DFT computational analysis indicated that the HOMO region was distributed over benzimidazole-pyrimidine and aniosole-N, whereas the LUMO was over the whole molecule and benzimidazole-pyrimidine for Q-NO_2_ and Q-OMe, respectively. On the other hand, by means of MD analysis, it was suggested that both molecules were adsorbed with a planar orientation, allowing optimal interactions between heteroatoms and *π* electrons and the Fe (110) surface. Hassan et al. [21] studied a derivate of the β-lactam ring, 2-(3-chloro-2-(4-hydroxy-3,5-dimethoxyphenyl)-4-oxoazetidin-1-yl)-1-methyl-1H-imidazole-4(5H)-one (COMI). The reported IEs were from 90.2 to 94.2% at concentrations ranging from 40 to 80 ppm at 323 K. Unlike the previous studies, the behavior benefitted from the increasing temperature, reaching IE values of 95.5, 97.4, 97.7 and 98.4% at 20, 40, 60 and 80 ppm, respectively, which was associated with the endothermic nature of the adsorption process, as well as to multiple heteroatoms located in the terminal groups of the structure, which favored adsorption on different simultaneous active sites.

In contrast, Alia Atiqah Alias et al. [35] analyzed the organometallic compound bis(N-ethyl-isopropyl dithiocarbamate)chlorido butyltin (IV) [EIDTC2ClBuSn]. The chemical structure was selected based on the presence of donor heteroatoms like S and N, as well as for its larger molecular size, which gave a wider surface coverage of the metal. The evaluation by potentiodynamic polarization (PDP) reported IEs from 96.9 to 98.3% at concentrations from 0.001 to 0.005 mM. The SEM analysis exhibited the diminution of the surface damage in the presence of a CI, whereas by EDX, the elements O, Cl, N, S and Sn were identified. It should be mentioned that the authors indicated that even with the obtained results, the compound adsorption was incomplete due to its non-planar structure, which could have prevented the observations of specific regions with high S and Sn percentages. The DFT analysis revealed that the HOMO was located over the ligand dithiocarbamate, specifically over S bound to Sn, whereas the LUMO was located over the ethyl-isopropyl chain surrounding the N of dithiocarbamate. Another study where larger chemical structures were employed was developed by Belkheiri et al. [36]; in this work, a derivate of xanthene 14-(4-nitrophenyl)-14H-dibenzo[a,j]xanthene (ZM6) achieved IEs between 91.3 and 92.2% at 1 × 10^−4^ and 1 × 10^−3^ M. Its behavior was associated with the presence of aromatic rings and the conjugated *π* system. Nonetheless, since the IE is a function of the medium temperature, it diminished from 92.2 to 88.1% when the temperature increased from 298 to 328 K. According to these authors, the xanthene structure provided steric stability, whereas the presence of a nitro group (–NO_2_) favored the dipole interactions. Additionally, the molecule was analyzed by DFT, describing a slightly curved structure between the group 4-nitrophenyl and the planar part in (14H-dibenzo[a,j]xanthene). On the other hand, the molecular orbitals (MOs) were clearly defined in the molecule, where the HOMO was located over part of xanthene, whereas the LUMO was over 4-nitrophenyl.

In the field of CIs, the idea of profiting from the use of drug active compounds, preferably of those that have expired, either by using their commercial forms or by separating them from their excipients, has emerged in order to support sustainability, handling of residues and to reduce costs and their impact on the public health [56]. In the first study, Li et al. [37] studied tetracycline and oxytetracycline, which achieved an IE of 91.3% at 2 × 10^−6^ and 5 × 10^−5^ M, displaying minimal response to slight temperature changes (293–323 K).

According to the authors, a competitive adsorption process occurred, implying both antibiotic molecules with the Cl^−^ ions present in the corrosive medium, favoring, in the first place, physisorption, which diminished the systems’ randomness and promoted further chemisorption. MD studies indicated that the hydroxyl groups in oxytetracycline facilitated the formation of intermolecular H bonds, suggesting the formation of a five-member ring that made difficult the transport of electrons to the Fe 3d orbitals; thus, diminishing its inhibiting effect, whereas tetracycline displayed better lateral distribution, which affected the competitive adsorption of tetracycline and Cl^−^ ions. As for the inhibition mechanism, four stages were suggested: (1) electrostatic attraction, (2) interaction with unshared electrons, (3) interaction between the metal and *p* electrons and (4) synergy implying the three previously mentioned stages.

In this context, Nouri et al. [38] studied the medicament ketoconazole (KETO), which was an effective CI of other metals such as aluminum and mild steel in the sulfuric acid medium. Like with the previously analyzed tetracyclines, temperature exerted a minimal effect on the KETO IEs, reporting that when the temperature increased from 296 to 316 K, the IE changed from 93.3 to 93.2% (50 ppm) and from 93.5 to 95.2% (100 ppm). The authors suggested that the molecules were protonated by the N atoms and adsorbed by means of electrostatic attractions on Cl atoms that were previously adsorbed on steel; on the other hand, neutral molecules were adsorbed by chemisorption parallel to the surface through the donation of *π* electrons in the aromatic rings and electron lone pairs from the heteroatoms. The DFT analysis revealed that the MOs were distributed in opposing molecule zones, where the HOMO was over the phenyl-piperazinyl-ethanone region and the LUMO was over dichloro-benzene.

Regarding Singh et al. [39], these researchers analyzed the inhibiting properties of expired atorvastatin (EA) as a financially viable and environmentally safe alternative for protection against metal corrosion. In comparison with other elimination or degrading methods that can be tedious, expensive and ineffective (just reaching partial drug degradation), the use of EA as a CI reduced its environmental impact by taking advantage of its functional properties instead of disposing of it. This compound achieved an IE of 94.4% at 50 ppm, displaying a minimal increase at higher concentrations. The authors suggested that the adsorption mechanism of EA occurred through three stages: (1) electrostatic attraction of the protonated molecule toward the metal surface negatively charged by the adsorption of Cl^−^ ions, which neutralized the metal, (2) chemisorption of the heteroatoms N and O with unshared electrons and (3) backdonation by means of Fe *d* orbital electrons transferred to the molecule *p* vacant orbital.

In recent years, ionic liquids (ILs), especially those with organic cations and anions, have drawn much attention because of their unique properties such as excellent thermal stability, high ionic conductivity, negligible vapor pressure, big electrochemical windows and low toxicity [57]. Tan et al. [7] analyzed the inhibiting properties of 1-hexadecyl-3-methylimidazolium bromide (HMIBr), reporting IEs of 94.5 and 96.9% at 2 × 10^−4^ and 1 × 10^−3^ M, respectively; such efficiency was associated with the replacement of water molecules adsorbed at the metal/solution interface by the cation and anion of [HMIBr]. By MD analysis, it was observed that the cation 3-methylimidazolium adopted a quasi-planar orientation over Fe (110), while the hexadecyl chain inclined toward the corrosive medium. On the other hand, Mobin et al. [40] studied an ionic surfactant based on a zwitterionic gemini IL, which is a compound that has simultaneously a positive and negative charge in the same molecule.

In particular, 2-decyl phosphate-1-(N,N-dimethyl,N-tetradecylammonium) ethane achieved IEs from 93 to 97.1% at concentrations ranging from 10 to 500 ppm at 298 K, although the temperature increase slightly affected the IE, reporting 92.3% at 50 ppm and 338 K. The authors ascribed the high IE values featured by the zwitterionic gemini surfactant to its chemical structure with ionic and non-ionic groups and the short length of the spacer group between the cation and anion. They could also be related to the charges in the cation ammonium and anion phosphate; furthermore, both ions include a relatively long alkyl chain (C_14_ and C_10_) that provided additional hydrophobicity to the CI.

The use of macromolecules has also been studied for mild steel, evaluating compounds such as copolymers [41] and functionalized graphene oxide [4] to natural biopolymers like poly-γ-glutamic acid (PGA) [42] and polypeptide chain (gelatin) [43]. In the area of copolymers, Souza et al. [41] studied a copolymer with relatively high viscosity (POPS) derived from polydimethylsiloxane (PDMS) and polyoxyalkylene (POA); this CI is widely known at an industrial level as a surfactant with thermal stability and low surface tension, and IEs from 93 to 97% at concentrations from 5 × 10^−6^ to 1 × 10^−3^ M at 298 K were reported. According to these authors, higher surfactant concentration can promote the formation of micelles, which diminishes the probability of replacing water molecules adsorbed on the metal surface. It was also suggested that the surfactant POPS was adsorbed chemically through the amine N atom (POA), orienting the PDMS main chain toward the solution.

In this context, Baig et al. [4] studied diethylenetriamine functionalized graphene oxide (DETA-GO) due to its hydrophilic character, which is in contrast with that of conventional graphene. In addition, the functionalization of epoxy, carboxyl, carbonyl and hydroxyl groups of the GO surface improves its solubility. IEs of 92.7 and 94.5% at concentrations of 25 and 50 ppm were reported, which were associated with higher surface coverage and availability of inhibiting molecules, suggesting that DETA–GO replaced gradually preadsorbed water molecules and formed an inhibiting film on the steel surface. In addition, the DFT analysis proposed that the HOMO was distributed mainly in the structure center, i.e., in the aromatic rings, whereas the LUMO was distributed over the phenyl rings and epoxide groups.

In the case of biopolymers, Roy et al. [42] analyzed the inhibiting properties of poly-γ-glutamic acid (PGA), which is a high-crystallinity-rigid polymer, insoluble in organic compounds with high strength and modulus [58]. An IE of 90.2% at 50 ppm and 293 K was reported; it is worth mentioning that the concentration had to be increased to mitigate the system temperature effects: at 200 ppm, an IE of 77.1% was obtained at 323 K. This behavior pattern suggested the dissolution of the polypeptide chain adsorbed on the metal surface due to its fragility in an acid medium. The authors suggested that PGA interacted with the steel surface by means of long-range electrostatic interactions through which the mutual partial transfer of charges could occur. On the other hand, the DFT analysis of the protonated PGA indicated that the HOMO was distributed over the amide group, whereas the LUMO was located exclusively over the carboxylic group adjacent to the terminal group NH_3_^+^. Likewise, the MD studies suggested that the protonated PGA form provided higher protection to Fe (110). Another study devoted to biopolymers was carried out by Pal et al. [43] who worked with ‘gelatin’, which is widely known as a heterogeneous mixture of single-stranded or multistranded polypeptides with non–uniform distribution of at least 18 amino acids. IEs of 90.5, 94 and 95.2% at concentrations of 25, 50 and 100 ppm at 303 K, respectively, were reported; in this case, it was also necessary to increase the concentration to keep the IE above 90% as the medium temperature increased. In contrast, gelatin kept an IE above 90% even after 72 h of exposure. According to the authors, the variation in ΔG°_ads_ originated from the competition between physical and chemical adsorption processes occurring on the surface. Also, a very interesting proposal was stated regarding the adsorption process occurring at multiple stages: (1) protonation of the chain amino acid, (2) electrostatic attraction toward the adsorbed Cl^−^ ions, (3) possible direct charge transfer (chemisorption) and (4) hydrophobic dehydration of the protein, provoking the opening of the protein chain, and then the freeing of more active sites (nonpolar amino acids) that can be adsorbed.

A very interesting structure was studied by Ituen et al. [26] who carried out the evaluation of the molecule N-acetyl cysteine (NAC) in the protection of mild steel and X80 steel in 15% HCl. IEs of 93.7 and 91.6% were achieved at 1.0 mM for mild steel and 0.5 mM for X80 steel, respectively. The functional groups thiol (–SH), carbonyl (–C=O) and amine (–NH–) played a major role in its behavior as a CI, with thiol standing out because of its high nucleophilicity and capacity to form coordinate bonds with metals, which allowed the creation of an effective protecting barrier. According to these authors, the carbonyl group provided stability through electronic and orbital interactions with the metal surfaces. On the other hand, amine can donate electrons by means of its nitrogen atom, although its IE tends to diminish if it is protonated in acid media. In comparison with other previously analyzed molecules, its size is compact, which gives advantages such as higher molecular mobility, adsorption facility, formulation flexibility, lower aggregation probability and specific chemical interactions that allow the right alignment and positioning on the metal surface.

Yadav et al. [6] studied the inhibiting properties of two compounds derived from thiazole, 1-(benzo[d]thiazol-2-yl)-3-chloro-4-(3,5-dichlorophenyl)-4-methylazetidin-2-one (BDMA) and 3-(benzo[d]thiazol-2-yl)-2-(3,5-dichlorophenyl)-2,5-dimethylthiazolidin-4-one (BDMT). In the case of BDMA, IEs from 90.3 to 95.4% at concentrations from 30 to 50 ppm were reported, whereas BDMT achieved IEs of 92.3 and 93.7% at concentrations of 40 and 50 ppm. These small IE differences can be related to the presence of a smaller ring and chlorine bond in the case of BDMA, which is in contrast with a five-member ring including S in the chemical structure of BDMT. However, the DFT analysis of the structure suggested that in both cases, the HOMO was located over the benzo-thiazol group, whereas the LUMO was distributed over the 3,5-dichrolophenyl ring and benzothiazole in BDMA and BDMT, respectively. The authors suggested that both CIs presented a considerable excess of negative charge in the heteroatoms (N, S and O), which favored the formation of coordination bonds between the CI and metal surface. In another study by the same research group [11], a less functionalized structure was analyzed, 2-(1-((piperazine-1-yl)methyl)-1H-benzo[d]imidazol-2-yl)-phenol (PzMBP), achieving an IE of 90.6% at 32.3 × 10^−5^ M and 303 K; such efficiency was attributed to the presence of four N atoms in the structure (benzoimidazole + piperazine), and possibly, also to an aromatic ring with an –OH group like phenol, which promoted the interaction of PzMBP through a physisorption/chemisorption combination. For this compound, the HOMO and LUMO regions were distributed over the benzimidazole ring and its adjacent phenol, which are rich in delocalized *π* electrons, thus suggesting that the piperazine ring did not contribute significantly to the adsorption process of PzMBP on N80 steel. Under the same experimental conditions, Wang et al. [13] analyzed the inhibiting properties of the IL imidazoline phenyl thiourea iodized n-butane quaternary ammonium salt (IMSQ), reporting an IE of 96.7% at 0.03 mM.

The results could be mainly associated with the physical and chemical adsorption of the cation functional groups, which improved the stability of the inhibiting film. Like in other studies, it was considered that the anion I^−^ worked as an intermediate bridge between the surface positive charge and the IL cation, and by covering higher surface area, it could displace Cl^−^ ions from the corrosive medium.

In contrast, Kousar et al. [44] studied a compound derived from imidazoline, (Z)-2-(2-(heptadec-8-en-1-yl)-4,5-dihydro-1H-imidazol-1-yl)ethan-1-amine (OMID), to diminish the corrosion of carbon steel, reporting an IE of ~96% at 0.01 mM. According to the results of the XPS analysis, the authors proposed a double single protonation of the structure: the first one in the primary amine group (NH_2_ → NH_3_^+^), and the second one in the imine group (>C=N → >C=NH^+^). Nonetheless, the tertiary amine group (N<) remained unprotonated. Similarly to other previous studies, it was suggested that the Cl^−^ ions participated as bridges between the surface positive charge and protonated molecule.

On the other hand, Shoair et al. [45] used expired terazosin to protect 1018 carbon steel. The weight loss (WL) tests revealed IEs of 91.4 and 92.3% at 50 and 75 ppm after 2 h of exposure, respectively. The authors proposed an inhibition mechanism that combined three processes (including the CI protonation): (1) physisorption through the molecule -NH^+^ toward the Cl^−^ ions previously adsorbed on the metal surface, (2) chemisorption through unshared electrons (–O–, =NH >NH) and (3) aromatic ring backdonation.

In contrast, for Q235 carbon steel, high IEs were reported by employing organic compound CIs derived from imidazole [5] and hydrazine [46], as well as by those derived from lignine [47,48], polymers [12] and gemini IL [1,49]. As for Lai et al. [5], these researchers studied a bromosubstituted nitrogenated heterocyclic compound, 2-(*p*-bromobenzylthio)-1H-benzimidazole (Br-BBD), reporting IEs of 90.6, 93.9 and 97.2% at concentrations of 40, 60 and 80 mg/L at 298 K, respectively. This behavior was associated with unshared electron pairs of the heteroatoms located in the benzimidazol and thioester groups, whereas Br played a minor role modifying the polarity and electronic density, as well as promoting the electrostatic interactions between the molecule and steel surface.

However, the authors analyzed the inhibiting properties as functions of different system variables: HCl concentration in the corrosive medium (0.5 M: IE = 99.74% and 3.0 M HCl: IE = 93.2%), corrosive medium teperature—CI (298 K: IE = 99.4% up to 338 K: IE = 94.9%) and storage time (0 h: IE = 99.4% and 196 h: IE = 99.1%), suggesting that Br-BBD displayed excellent results even under different conditions.

Luo et al. [46] analyzed the inhibiting properties of long chain fatty hydrazides (LCFHs) obtained from canola oil. This compound achieved IEs from 90.9 to 94.5% at concentrations from 10 to 50 ppm. According to these researchers, the LCFHs presented a combined mechanism of physical and chemical adsorption related to the presence of the active amide functional group that, through the N atom, can form coordination bonds by means of its unshared electron pairs. In addition, hydrazines are characterized by being antioxidant agents, which allows the diminution of oxidant agents such as solution O_2,_ minimizing its effect participating in corrosion reactions [59].

Regarding Liao et al. [47] and Luo et al. [48], these authors decided to analyze compounds derived from one of the most abundant and renewable resources in nature: lignin. Its inhibiting properties are due to its tridimensional reticular structure, and mainly to the presence of methoxy, phenolic, hydroxyl and carbobyl functional groups [60]. In the first study, for sodium lignosulfonate (SLS), IEs from 96.2 to 98.7% were reported at concentrations from 10 to 50 ppm, whereas in the second study, methacryloyloxyethyl trimethyl ammonium chloric (DMC) was added to the structure of nano-lignin (SLS nanoparticles), achieving IEs that were similar to those recorded for SLS (96.5% at 8 mg/L), suggesting that the addition of DMC did not significantly improve the IE under the evaluated experimental conditions, which can be associated with the limited synergy between the CI parts or with the surface CI saturation. The authors agreed with the fact that the formation of complexes between the lignin benzene ring and Cl^−^ and H^+^ ions in the corrosive medium can occur, whereas the sulfonate part was oriented toward the solution core, forming a dense film. In contrast, DMC-NL also possesses hydrophilic properties, which would allow its spontaneous adsorption through electrostatic interactions between the N positive charge and metal surface negative charge, as well as by unshared electrons of N (sp^2^) with *d* orbitals of the Fe atom. In addition, the researchers suggested that interparticle interactions and solid-state reactions can happen due to intermolecular forces and electrostatic interactions.

Liu et al. [12] studied the compound UPy-containing Poly(amino ester) (UPy-D400-PEGDA), which is a polymeric CI synthesized by the Michael addition reaction between polyether amine grafted 2-ureido-4[1H]-pyrimidinone (UPy-D400) and poly(ethylene glycol)-diacrylate (PEGDA). This polymer achieved an IE of 91.4% at 50 ppm, which was related to diverse functional groups such as heteroatoms (like N) and especially to O from pyrimidinone (possible chemical interaction), as well as to the linear and grafted polymeric skeleton. According to the authors, the protonation of N atoms facilitated their orientation toward preadsorbed Cl^−^ ions due to the charge difference.

In recent decades, an increasing trend featuring research works on ILs has been identified mainly because of their versatility and efficiency. In this field, gemini-type ILs have been evaluated, i.e., two-monomer conventional ILs linked by a spacer, which is, in general, an alkyl chain without functional groups. Particularly, Mao et al. [1] studied the compound N1, N1, N4, N4-tetramethyl–N1, N4–bis(2-(hexadecanoyloxy)ethyl)butane-1,4-diammonium bromide (TBDB). The selection of this CI was based on the presence of two groups with a hydrophilic head and of two groups with a hydrophobic tail per molecule, as well as on its surface activity, solubility and higher IE than in conventional ILs. In addition, it is worth mentioning that the addition of the hydrolyzable group hexadecanoyloxy improved the inhibitors’ biodegradability. This compound achieved IEs of 94.6, 98.1 and 98.4% at concentrations of 20, 40 and 60 ppm, respectively, without having to add a synergistic agent such as sodium salicylate (SS) or sodium iodide (NaI). As for the inhibition mechanism, it was suggested that since the distribution of the LUMO and HOMO was over bromine atoms and ester groups, respectively, the adsorption process of TBDB could proceed through the IL head with the long alkyl chain perpendicular to the metal surface, which resulted in a dense and ordered film. In this context, Fang et al. [49] evaluated the corrosion inhibiting properties of a halogenated IL derived from naphthyridinimides: 3,3′-(1,3,6,8-tetraoxo-1,3,6,8-tetrahydrobenzo[lmn][3,8]phenanthroline-2,7-diyl)bis(N-ethyl-N,N-Dimethyl-1-propylamine)bromide (NDI-N-Br). The authors reported an IE of 90.3% at 50 ppm, which was associated with the synergy between the anion Br^−^ and the diverse cation functional groups that facilitated the almost planar adsorption of the structure, as observed in the MD study. The DFT analysis revealed that the HOMO and LUMO were located over the anion and tetrahydrobenzo, respectively, suggesting that these were the main adsorption centers of the compound NDI-N-Br.

In the case of C38 steel, a study by Molhi et al. [50] was found; these researchers analyzed the inhibiting properties of an epoxy resin with chelating properties: pentaglycidyl ether pentaphenoxyphosphorus (PGEPPP). An IE of 93.2% at 0.1 mM was reported. This inhibiting behavior could be related to the presence of highly reactive functional groups such as epoxide and phenoxy-phosphorated groups, *π* electrons from aromatic rings and P unshared electrons. The DFT analysis suggested that the HOMO was over the benzene rings, whereas the LUMO was distributed through the entire molecule.

On the other hand, two studies on carbon steel were found: one was based on thiocarbohyrazides-dibenzamide [51] and the other on a halogenated IL derived from pyridinium [52]. In the case of the first study, two compounds were reported: N,N′-[2,2′-thiocarbonylbis(hydrazine-2,1-diyl)bis(thioxomethylene)]dibenzamide (T1) and N,N′-[2,2′-thiocarbonylbis(hydrazine-2,1-diyl)bis(thioxomethylene)]bis(4-methoxybenzamide) (T2). For T1, an IE of 92.7% at 1 × 10^−4^ M was reported, whereas for T2, the IE was 95.9% at 5 × 10^−5^ M. This behavior was mainly associated with the presence of multiple adsorption centers in different functional groups with N and S. Notwithstanding, the small IE difference could be related to the addition of the terminal group methoxy, which boosted the interaction between *p* electrons and the empty Fe *d* orbitals. In this context, Öztürk et al. [52] analyzed the inhibiting properties of an IL derived from functionalized pyridinium: 1-[3-oxo-3-[(4-oxo-2-phenylquinazolin-3(4H)-yl)amino]nonil]pyridinium bromides (**3c**). This compound kept its IE of ~93% at concentrations ranging from 10 to 150 ppm, which was attributed to the alkyl chain (C_9_) and a polar unit with multiple adsorption active centers that included oxo, phenylquinazolin and amino groups, which can work synergistically by donating unshared electron pairs (N and S heteroatoms) and aromatic ring *π* electrons to the steel surface. Regarding the mechanism, the adsorption of Cl^−^ anions on the positively charged steel surface, which provoked the electrostatic attraction of the cation, was considered as the first stage. In this case, the anion Br^−^ could also contribute to the modification of the steel surface charge. Furthermore, the authors suggested that quinazoline derivatives can be protonated like in other previously analyzed studies.

In another study, Nouri et al. [53] evaluated the protection of steel by employing the antifungal Fluconazole. The authors reported IEs from 92.4 to 90.5%, varying the temperature from 296 to 316 K at 50 ppm. This inhibiting property could be associated with the following factors: (a) presence of triazole groups, which contributed to the formation of coordination bonds between lone electron pairs and the vacant Fe *d* orbital, (b) hydroxyl groups, considered as polarity modifiers, and O and N heteroatoms, related to electron transfer to metal surfaces by chemisorption and/or backdonation), and (c) the F atom that provided the hydrophobic character to the molecule part it was bound. The DFT analysis of the neuter and protonated structure revealed that the HOMO was located over difluorophenyl, whereas the LUMO was over difluorophenyl and triazole.

Some compounds stand out among the studies with the highest corrosion IEs in a HCl medium at concentrations equal to or greater than 1 M, with efficiencies greater than 90% at concentrations lower than 17 ppm. These ones include derivatives of pyrimidinium, thiourea, quinazoline, thiourethanes, imidazole, lignin, antibiotics and phosphates [29,32,34,35,37,40,44,48,52]. The authors of these studies reported similarities in the chemical structures and inhibition mechanisms of these molecules, which improved their ability to reduce corrosion at minimal concentrations. These mechanisms include the protonation of compounds in the HCl medium, which facilitates their dissolution and subsequent adsorption on the metal surface through heteroatoms [29,32,34,44,52]; the presence of functional groups containing heteroatoms such as O, S or N [29,32,37,48,52]; the ability to donate and receive electrons from aromatic rings [29,34,48,52]; chloride ions in the acidic medium acting as bridges between the surface positive charge and the CI [32,44,52]; planar molecular orientation [32,34,35,37]; electrostatic adsorption followed by the formation of coordinate bonds with the metal [32,37]; and relatively long alkyl chains (C_9_ and C_14_), which provide hydrophobicity to the CI [40,52].

#### Structure-Efficiency Relationship of CIs in a HCL Corrosive Medium

The analysis of organic CIs with IEs above 90% in a HCl medium revealed that there was no direct correlation between the structural complexity and inhibiting performance. Compounds with simple structures such as thioureas or aromatic amines have achieved IEs comparable to those reported for more complex derivatives with multiple functional groups, long alkyl chains or extended conjugated systems. Even when the frequent presence of heteroatoms such as nitrogen, oxygen and sulfur, as well as aromatic rings and heterocycles have been observed, it seems that the IE depends more on the electronic orientation, effective adsorption capacity and compatibility with the corrosive medium than on the absolute number of donating groups. This structural diversity among the most effective compounds suggests that the interaction with the metal surface can be governed by mixed physical and chemical adsorption mechanisms, where factors such as polarity, localized electronic density and molecular flexibility play a defining role.

### 2.2. Corrosion Inhibition of Carbon Steel in Sulfuric Acid

Corrosion by sulfuric acid is more common in sulfuric acid alkylation facilities and treatment plants of residual water, where there is not meticulous control of the acid concentration, flow rate operative temperatures and acid flow [61,62]. The transport of acid through devices and pipeline systems that were not designed to withstand acid surroundings can provoke an increase in corrosion indices [63]. Reactor effluent lines, reheat systems, deisobutanizer heads and heating treatment systems are especially vulnerable, whereas the containers that are in contact with sulfuric acid can also undergo severe corrosion when cleaned as part of internal checking [64,65,66,67].

Corrosion damage of carbon steel by sulfuric acid is widely known and it occurs as general corrosion (uniform) or localized corrosion (by pitting). General corrosion attack is the beginning of the steel corrosion process; this term is also used in cases where damage is widely extended and the corrosion level is not extreme in a specific surface region, whereas localized corrosion by pitting stands out because of its grave degradation effects on the integrity of the steel metallic structure [68].

The corrosion mechanism by sulfuric acid, like the one with HCl, also involves anodic and cathodic electrochemical reactions that are described as follows: first, H_2_SO_4_ in solution dissociates through two stages, forming two protons (*H^+^*) (Reactions (11) and (12)) [69]. When carbon steel comes into contact with dilute sulfuric acid, the immediate attack of the metal, and simultaneous formation of hydrogen (hydrogen evolution or gaseous hydrogen production) (Reactions (8)–(10)) [70] and ferrous ions (metal anodic dissolution (Reactions (13)–(15)) occur; it is worth mentioning that the iron oxidation reaction takes place in the ferrite phase, whereas the hydrogen cathodic reduction reaction occurs in the cementite phase [71].

*First dissociation*

(11) $$H_{2}SO_{4}\to HSO_{4}^{-}+H^{+}$$

*Second dissociation*

(12) $$HSO_{4}^{-}↔SO_{4}^{2-}+H^{+}$$

Afterward, the anodic dissolution of iron proceeds through three stages:

(13) $$Fe+SO_{4}^{2-}\to {\left(FeSO_{4}^{2-}\right)}_{ads}$$

(14) $${\left(FeSO_{4}^{2-}\right)}_{ads}↔{\left(FeSO_{4}\right)}_{ads}+2e^{-}$$

(15) $${\left(FeSO_{4}\right)}_{ads}\to Fe^{2+}+SO_{4}^{2-}$$

Iron oxidizes with the formation of ferrous sulfate (*FeSO_4_*)*_ads_*, which adheres to the steel surface and forms a protective layer that prevents the metal from being attacked by the acid environment. Notwithstanding, it is weak and slightly adherent, which means that the metal surface can be damaged easily and significantly accelerates its corrosion process, in addition to other important factors such as FeSO_4_ solubility in the acid medium, temperature and relative movement between the metal and acid [72,73].

The uniform metal loss from the pipeline walls is mainly due to the anodic dissolution of iron. The rate of this corrosion type can be predicted; then, if the pipeline general thinning rate is calculated correctly, it can be mitigated by adding corrosion tolerance to the thickness during the design phase or chemical treatment during its functioning [74]. It is worth mentioning that the corrosion rate of carbon steels in dilute acids depends mainly on the steel chemical composition, especially on the carbon content. Likewise, the formation of a protecting film of corrosion products can condition the steel corrosion rate and is defined by oxidant diffusion (H_2_SO_4_ in this case) through the layer of corrosion products (FeSO_4_) and reaction rates of the corrosion process or diffusion rate of the corrosion products from the metal surface toward the solution [73].

Table 2 shows a list of steel CIs in H_2_SO_4_, where it is remarked that the addition of 100 ppm or less of these CIs can achieve an IE ≥ 90%. It can be observed that they feature diverse functional groups such as pyridine [64,67], pyrimidine [75,76,77], triazine [78], triazole [15,79,80], phenylchromene [81], benzenesulfonamide [17], benzimidazolyl [82], pyrazolone [83], non-ionic-surfactant-based [84,85], sulfonate [86], imidazolium–based ILs [63,87,88] and ammonium [89,90], as well as some drugs [16,91]. In general, the CIs for steels exposed to sulfuric acid corrosive media are chemical compounds consisting of S heteroatoms (C–S–C, C=S, –S=O, S–NH_2_, –SH), O (C=O, –OH, C–O–C) and N (C–N, N–N, –C–NH, N–R, ≡N, –N=, –NH_2_), as well as of three-, five-, and six-member rings that can include resonant or single bonds.

Some researchers have exploited such contributions by synthesizing polymers or by adding longer alkyl chains (>C_10_) to keep suitable IEs even at low concentrations in comparison with ‘smaller’ inhibiting molecules. In the case of El-Lateef et al. [67], these authors synthesized the ionic polymer AMTP, which combined the properties of dicarbonylpyridine and dihydrazine-thiazole to mitigate the corrosion of 1018 carbon steel in 1.0 M de H_2_SO_4_. In the case of hydrazine, it was selected because it can provide stretching, resistance and ionic conductivity properties. In contrast, thiazole was added to increase antimicrobial action and the polymers’ thermal stability. According to these authors, the polymer AMTP can be synthesized with relatively economical raw materials, achieving good yield and high solubility. The results of the electrochemical techniques suggested that by occupying a larger area than water molecules and aggressive ions, characteristic of the medium, the displacement of molecules by the adsorption of the polymer AMTP was possible. Furthermore, the molecular simulation of an AMTP monomer reported that the electrophilic and nucleophilic attack regions were located in the hydrazine, pyridine and thiadiazole groups.

In contrast, Abdulridha et al. [64] studied the organic molecule AS, based on pyridine functionalized with multiple N heteroatoms and aromatic rings such as benzonitrile and phenyl, to protect steel carbon. The selection started with Schiff bases linked through –C=N– bridges connecting heterocycle, conjugated, flat and electron rich compounds with certain functional groups, adding imine bonds (RR′–C=N–R″) with functional groups such as hydroxyl, thiol, carbonyl and N heteroatoms, which eased the formation of coordination bonds with the steel surface. By means of electrochemical tests, it could be seen that the IE had a direct relationship with the analyzed concentrations; on the contrary, an inverse relationship with the increasing temperature was observed. Also, the adsorption/desorption rate affected the IE. In particular, AS displayed standard Gibbs free energy characteristic of mixed adsorption; however, it tended mainly toward chemisorption, which was associated with the formation of coordinate bonds due to the exchange of charge and/or transfer of electrons between the molecule molecular orbitals and Fe *d* orbitals. The DFT analysis found that the aromatic rings and N and O bonds (–CN, –N=N–, –C≡N–, –OH) were related to the electron donating capacity, whereas the electron accepting feature of the metal surface was distributed through the whole molecule, except in the pyridinium ring attributed to the presence of the CN group.

In the case of Hameed et al. [75], these researchers employed the organic compound C3 consisting of pyrimidine, pyrazole, phenyl and hydrazide for the same metal and corrosive medium. A linear relationship between the surface in the absence and presence of C3 was reported, suggesting the absence of an insoluble film of corrosion products and/or CI on the metal surface, which was associated with the CI that could work through two ways: by blocking surface active sites or by modifying the cathodic and anodic reaction mechanism. Like the previous study, it was found that temperature affected the inhibitor behavior, making the film less stable and provoking its desorption, which increased the surface damage by freeing new reactive metal sites. By analyzing the temperature effect on the IE, a direct relationship between the temperature and activation energy (E_a_) was identified, proposing that with the increasing temperature, the desorption was intensified and then, more energy related to higher corrosion rates occurred because of the increasing effective area exposed to the corrosive medium.

The use of resins is a not common proposal, like the one by Hsissou et al. [78] who synthesized the epoxy resin macromolecule ERT, based on triazine functionalized with amine/oxirane groups, by means of three-step condensation reactions to protect carbon steel in 0.5 M H_2_SO_4_. The electrochemical impedance studies suggested a direct relationship among the CI concentration, the diameter of the semicircles in the Nyquist plot and thickness of the inhibiting film formed on the steel surface, which was atributed to the adsorption of the CI on the active centers and its replacement of aggressive molecules, occupying a higher area and diminishing the contact between steel and the corrosive medium. This behavior pattern was associated with the presence of heteroatoms and heterocycles with nitrogen and oxygen, as well as of oxirane groups. Like in the previous cases, the temperature increase provoked a higher desorption rate of ERT without modifying the adsorption mechanism. On the other hand, the authors suggested that first, the sulfate molecules (SO_4_^2−^) are adsorbed electrostatically on the metal cathodic zones, producing the further attraction of the protonated ERT form and promoting its replacement on the active sites and adsorption on the anodic sites. Furthermore, ERT can be adsorbed through its heteroatom unshared electron pairs. Based on the DFT analysis, it was found that the triazine ring and the three central amine groups had the capacity to donate electrons to the empty iron orbitals, whereas the carbon atoms bound to amine groups were capable of accepting iron electrons through the backdonation process.

Another widely studied group is that of heterocycles with nitrogen atoms such as triazole, mainly the compound 1,2,3-triazole studied as a CI of diverse metals like steel, copper, iron and aluminum, among other alloys. These compounds and their functionalized derivatives are obtained by “click chemistry reactions”, which feature advantages such as simplicity, effectivity, high yield and stereospecificity [92]. These compounds have also shown biological activity, mainly antifungal and antimicrobial [93]. In this field, Elazhary et al. [79] synthesized and studied the compound MBTTA, based on triazole functionalized with benzoamide, acetate and tolyl, to protect sweet steel. By means of EIS tests, the increase in charge transfer resistance was observed, which resulted in higher IEs due to the progressive replacement of water molecules by MBTTA ones in the metal-solution interface. On the other hand, the CI response to temperature was analyzed, finding that its increase promoted a higher desorption rate of molecules, which was associated with the predominant physical adsorption of the compound and the concomitant IE diminution. The DFT analysis of the structure found that the distribution of the HOMO was located on the triazole ring nitrogen atoms, whereas the LUMO was over the structured carbon atoms.

In contrast, Saini et al. [81] preferred to study the inhibiting effect of the compound flavoxate, commonly known as urispas, which is a medicament based on chromene functionalized with piperidyl and carboxylate. From the electrochemical tests, the authors suggested that when adsorbed, urispas blocked the active sites on the metal surface by means of oxygen nonbonding electron pairs and ring *π* electrons. Similarly to the previous cases, its IE was affected by the temperature increase. During the DFT analysis, the molecule displayed the thorough polarization of the HOMO and LUMO, located over the piperidyl ring and the rest of the molecule, respectively. By AFM, it was observed that urispas promoted the formation of an insoluble film that decreased the surface damage and then, heterogeneity itself.

On the other hand, pyrimidines are heterocyclic aromatic organic compounds with nitrogen atoms at positions one and three of the six-member ring. The functionalization of the ring of nitrogen atoms and C_2_/C_4–6_ carbon positions provides a big number of possibilities, including high biological activity and farmacological, which according to the literature, set them as environmentally friendly compounds.

For this reason, their application as CIs has grown in recent years, in addition to their capacity to donate electrons from the Fe unoccupied *d* orbital through covalent/coordinate bonds and the reception of free electrons from the metal by means of backdonation [94]. This family of compounds was studied by Soltani et al. [77] who synthesized and evaluated PTM and PTMO, which are compounds derived from pyrimidine–thiols. From the results of the WL and EIS techniques, a diminution of the surface damage at low concentrations was observed, which was associated with the formation of an isolating adsorption layer on the steel surface and that reduced the charge transfer from the metal to the solution. The authors proposed that PTM and PTMO worked through a geometrical blocking process without altering the metal dissolution mechanism, promoting the inactivation of certain regions on the metal surface with respect to the corrosive solution. Notwithstanding, these compounds were sensible to the increasing temperature, diminishing their IEs in 25–30% at temperatures from 273 to 338 K. By DFT analysis, the HOMO was located mainly over the pyrimidine-thiol ring, being sulfur the atom with higher contribution. In contrast, the LUMO was distributed around the whole molecule, which suggested no specific interactions (no covalent) with the metal surface. On the other hand, dynamic simulation revealed the planar adsorption of the compounds on iron atoms, which supported the reported IEs when maximizing the area occupied on the metal surface.

Other known important groups are those derived from benzene. These compounds stand out due to their aromaticity, i.e., a cyclic structure that contains delocalized electrons that give stability and allow interactions with metal surfaces [95]. An illustration of this is the work by Xu et al. [17] who synthesized a compound derived from *β*-aminoalcohol (TDB) to protect sweet steel. According to the literature, amino alcohols can significantly diminish the anodic dissolution of sweet steel. Likewise, it has been considered that the addition of rings and functional groups such as –NH and –OH can improve the properties of an organic inhibitor. This compound achieved IEs within an interval ranging from 87.9 to 94.6% at concentrations from 5 to 50 ppm after 12 h of immersion in H_2_SO_4_ solution.

The researchers indicated that the presence of multiple functional groups featuring N, O and S contributed to the adsorption process through physisorption (H_3_NO_2_S) and chemisorption (–NH, –OH, –O–, benzyl ring) processes. Particularly, the DFT analysis revealed that the HOMO was located at the benzenesulfonamide rings, whereas the LUMO was found in the alkyl region between the two –NH groups. In addition, the combination of three molecule interactions was suggested: (1) the protonated form of TDB was adsorbed electrostatically on the cathodic sites of the metal surface, (2) the heteroatoms were adsorbed by means of coordination bonds with Fe vacant *d* orbitals and finally, (3) the aromatic ring was adsorbed chemically through donor–acceptor interactions between the *π* electrons and iron vacant *d* orbitals.

Another important group is represented by azoles, which include N-azole, N/O-azole and N/S-azole compounds that are five-atom aromatic rings consisting of N and another equal or different atoms such as sulfur or oxygen as part of the ring conjugated with the azole structure. In addition to featuring heteroatoms, double bonds and a flat structure, azoles are soluble in almost any polar medium, and for this reason, their application does not present so many limitations like that of other compound families. Furthermore, azoles have displayed wide biological activity such as antifungal, antidiabetic, immunosuppressant, anti–inflammatory, antiviral, antituberculosis and anticarcinogenic, as well as oral bioavailability and chemical stability [96,97].

Based on the foregoing, El-Aadad et al. [82] studied 2,2′-dibenzimidazolyl butane (DBIB), which is an aromatic compound that results from the combination of 1,3-imidazole and a benzene ring. The authors based their selection on the acid and basic characteristics of the –NH group. The compound achieved an IE of 92% at 50 ppm; however, it diminished with the increasing concentration, which was attributed to the weakening of the metal–CI interactions by the replacement of either water molecules or sulfate ions by CI molecules, which in turn could be associated with steric repulsion processes caused by the increasing number of available molecules to be adsorbed interfering with each other during their adsorption process. On the other hand, the compound displayed significantly high thermal resistance by reducing its IE by just 7% with a temperature increase of 30 K.

Another compound derived from the azole family is triazole, which consists of a five-member ring with three nitrogen atoms. In the literature, these compounds are considered to be environmentally friendly due to their high chemical activity and low toxicity. In addition, the interest in this compound as a CI stems from its amphoteric nature that enables it to form acids and salts, as well as from its affinity with metals due to its abundant *p* and nitrogen unshared electrons that allow the displacement of water molecules [98].

In this context, Hazazi et al. [15] studied a triazole molecule functionalized with methyl, amino and thiol (AMTT) groups to protect sweet steel. In this work, IEs of 92 and 94% at concentrations of 20 and 25 × 10^−6^ M, respectively, were reported, and the surface protection process was associated with simple blockage, where the inhibitor adsorption did not change the corrosion mechanism. Notwithstanding, AMTT featured an IE decay of 14% with an increase in temperature from 293 to 323 K. On the other hand, the authors suggested that the molecule was adsorbed by the combination of physical and chemical processes.

Another azole derivative, less frequently used as a CI in H_2_SO_4_ medium is pyrazolone. This organic compound is characterized by having two adjacent nitrogen atoms and a carbonyl (C=O) group, and is very valued in combinatory and medical chemistry due to its simple preparation and wide biological activity [99]. This type of compound was studied by Zhang et al. [83] who evaluated the inhibiting properties of PAP, which is pyrazolone functionalized with phenyl and amine. The authors found that its addition to the corrosive medium diminished steel damage from 95.1 to 98.7% at concentrations from 0.05 to 1 mM, which was related to the fact that the CI modified the cathodic and anodic reactions occurring on the metal surface through physical and chemical adsorption of the CI, forming a protecting film that blocked the metal active sites and prevented the attack of sulfate ions.

On the other hand, a surfactant is defined as a substance or a mixture of substances that reduces the surface tension between a liquid and a solid. Its unique molecular structure consists of two parts: (1) hydrophilic head and (2) hydrophobic head, i.e., these compounds feature a dual nature, where the hydrophilic part interacts with polar molecules such as water and other ions, whereas the hydrophobic one interacts with other hydrophobic substances such as hydrocarbons. Their application as CIs is due to several reasons, such as their capacity to form protecting films, modify the water-metal interface and repel water molecules, but their aggregation and micelle formation capacity stands out as their main advantage as CIs to protect metal surfaces from corrosion [100,101]. For this type of inhibitor, there is a wide variety of commercial products that can be employed alone or synergistically in combination with other CIs due to the fact that they can optimize the dispersion of the CI in the solution and increase the surface coverage range [102,103].

In this context, Branzoi et al. evaluated some members of two families of non–ionic surfactants, Triton [84] and Tween [85], both for protecting OL37 carbon steel. In their first study, the researchers evaluated Triton X-100 and Triton A-20, reaching IEs of 94 and 91% at 50 ppm, respectively. According to these results, these CIs presented high thermal stability, which diminished their tendency to desorb from the steel surface. In both cases, the structures feature a benzyl group and functionalizations with O; in the case of Triton X-100, the molecule features a longer alkyl chain, which explains its effectivity, whereas Triton A-20 presents a mixture of compounds with O heteroatoms, being mainly associated with 4-(2,4,4-trimethylpentan-2-yl)phenol that also includes a benzyl ring. In their second study, Tween 60 reached an IE of ~92% at concentrations ranging from 20 to 50 ppm, whereas Tween 80 achieved IEs from 87 to 94% at concentrations from 20 to 50 ppm. Both CIs are polyoxyethylene derivatives of the product Span, which boost their inhibiting properties by adding oxygen heteroatoms that favor the adsorption and formation of an inhibiting film that prevents the penetration of aggressive ions to the metal surface. The four evaluated CIs presented Gibbs free energy values characteristic of combined physisorption and chemisorption processes, mainly associated with the presence of an oxygen heteroatom; in the case of Triton X-100 and Triton A-20, both compounds consist of aromatic rings that can improve the adsorption capacity. As for Tween 60 and Tween 80, these molecules feature long alkyl chains, which provide hydrophobicity to the surfactant. In another study by the same author [86], an ionic surfactant called SSD achieved an IE of 93% at 50 ppm. The research work suggested that the surfactants modified the metal/electrolyte interface, forming a film that limited the contact between the metal and the solution. Another important factor was that the highest IE was achieved at concentrations higher than the critical micelle concentration (CMC).

Regarding the corrosion inhibition of Maraging steel, an efficient study was found, which was performed by Mary et al. [80] who evaluated two Schiff bases derived from triazole functionalized with thiophene (MTATT) and amino/benzylidene (DBAMTT) in a combined HCl and H_2_SO_4_ medium. These CIs were selected because of the presence of nitrogen and sulfur atoms, double bonds and aromatic rings that complemented the Schiff base. Unlike what was expected, these compounds exhibited an increase in IE as a function of concentration and temperature, achieving values ranging from 95.1 to 96.9% at concentrations from 10 to 75 ppm and from 94 to 97.7% at concentrations from 5 to 50 ppm for DBAMTT and MTATT, respectively. The Gibbs free energy values of the adsorption process of these CIs indicated that a chemisorption process took place, which supported the previously described behavior pattern. In the mechanism, the presence of the CI protonated species was emphasized, which was located on the imine nitrogen, and was attracted electrostatically to the negative region of the metal surface, whereas the neuter CI species occupied active sites by displacing water molecules and carrying out chemisorption by means of donor–acceptor interactions between ring *π* electrons and unpaired heteroatom electrons and iron vacant *d* orbitals. On the other hand, the DFT analysis revealed that the HOMO and LUMO were distributed over most parts of the molecule, except in the case of MTATT, where the LUMO was mainly distributed in the triazole ring and thiol group.

In the case of API steels, a research work by Espinoza-Vázquez et al. [76] was found, where the compounds MTBU-I and MTBT-Br, structures based on pyrimidine/triazole with different halides, were studied. The CIs reached IEs of 93.6 and 95.4% at 50 ppm, respectively; such behavior was associated with the adsorption of the structures on the metal surface, displacing and replacing water molecules and other previously adsorbed ions. Particularly, both CIs presented characteristics of physisorption combined with chemisorption, the latter being the predominant one. The authors emphasized the importance of incorporating an electronegative halogen atom into the CI structures to achieve high IEs by powering other functional groups such as triazole, pyrimidine and phenyl. On the other hand, the structures featuring bromine and iodine were more efficient because the halogen effect depends on the size, ionic charge, ionic radius and electronegativity. According to what has been observed in the literature, the presence of halogens also contributes to a higher availability of electrons, which facilitates the adsorption process by being coordinated with Fe *d* orbitals. Furthermore, the addition of a methyl group to the original structures without halogens slightly increased the IE depending on the added halogen. Also, the effect of flow rate and time on the IEs reached by the CIs were analyzed, reporting that MTBU-I (IE_40 rpm_ = 87.5%; IE_72 h_ = 91.2%) displayed the best performance in comparison with MTBT-Br (IE_40 rpm_ = 81.4%; IE_48 h_= 90%), which was related to a more compact inhibiting film and possibly to the halogen electronegativity (I > Br).

In the field of CIs, the cations that generally stand out are nitrogenated ones such as imidazolium and ammonium because structural modifications of their precursors (imidazole and ammonia) provide higher capacity to be adsorbed on metal surfaces and, as a consequence, to inhibit corrosion.

In the case of imidazolium, Corrales-Luna et al. reported IEs of 90 [87] and 93.1% [88] for the compound 1-Ethyl 3-methylimidazolium thiocyanate [(EMIM)^+^(SCN)^−^] tested in the corrosion inhibition of API X52 steel. (EMIM)^+^(SCN)^−^ was adsorbed on the metal surface, displacing water molecules and ions, which limited the contact between the metal and acid solution, thus keeping a more homogeneous metal surface. This inhibiting behavior was associated with the synergy between the IL cation and anion, where (EMIM)^+^ covered a larger surface area by means of backdonation (ring *π* electrons) and coordination bonds (N free electrons) with iron vacant *d* orbitals. Also, the small size of the anion (SCN)^−^ enabled it to occupy the free spaces with a positive charge on the metal surface. Another study of an IL derived from imidazolium was carried out by Elkholy et al. [63] who employed a gemini IL based on hexadecyl imidazolium bromide (GS) to protect carbon steel, reporting an IE of 90.5% at 50 ppm. By means of PDP and EIS electrochemical tests, it was suggested that GS did not change the corrosion mechanism and that by increasing its concentration, the relocation of previously adsorbed molecules occurred, passing from planar adsorption to a vertical one, which allowed higher coverage by cation piling, thus increasing the thickness of the inhibiting film. The authors suggested three adsorption processes: (1) through ring *π* electrons, (2) or through lone N electron pairs and (3) by interactions between the full orbitals of surface iron atoms and heteroatom vacant orbitals (backdonation). It should be noted that the previous interaction modes could occur simultaneously.

The use of quaternary-ammonium-based ILs has also been analyzed by two authors. In the first study, Abd-El-Naby et al. [89] selected cetyltrimethylammonium bromide to protect carbon steel, reporting an IE of 92.25% at 0.2 mM and 303 K. According to the electrochemical tests, the authors suggested that the CI molecules were adsorbed on the steel surface forming an adherent film with higher volume than the one consisting only of corrosion products; such behavior could be ascribed to the volume occupied by the CI cation. It was also considered that CI adsorption was integral (physical and chemical) and ideal (without mutual interactions between surfactant molecules in the metal/solution interface). In particular, the CI cation was adsorbed on the steel surface from its hydrophilic head (ammonium), leaving its hydrophobic part toward the solution core (C_14_H_29_ alkyl chain), whereas the anion Br^−^ worked as a bridge between the metal positive regions and the cation ammonium, thus facilitating the formation of an inhibiting film on most parts of the metal surface. Another analyzed variable was time, finding that the CI kept its IE at 96.2% after 2 h of immersion, which was related to the complexity of the adsorption process.

In this context, Olivares-Xometl et al. [90] synthesized and studied a group of halogen–free–ammonium–based ILs. From the four analyzed ILs, just one reached an IE of 91% at 75 ppm: DMEC_16_N-ES. The results were associated with the length of the cation and anion alkyl chain, which boosted the IL hydrophobic effect, forming a barrier that partially blocked the active sites formed by steel surface imperfections and inclusions. The electronic density of the cation and anion heteroatoms, which was oriented toward the metal surface, allowed the hydrophobic alkyl chains to be located toward the solution, thus preventing the diffusion of water molecules and aggressive ions. The authors emphasized the importance of the alkyl chain in CIs and suggested that long chains do not always increase IE values due to the possible packaging process of alkyl chains. This important observation differs from the generalization of designing CIs with long hydrocarbon chains. Likewise, it was stated that multiple, longer alkyl chains in the hydrophilic part (N) can also limit their interaction with the metal cathodic sites.

In contrast, Zhou et al. [91] employed omeprazole (OP), a benzimidazole–derived medicament that is highly commercially available and known as an inhibitor of the proton pump, to mitigate the corrosion of Q235 steel in a dilute medium of 0.1 M de H_2_SO_4_. Its evaluation as a CI reported an IE of 96.31% at 5 ×10^−5^ M, and EIS analysis revealed that the charge transfer process was driven by the interfacial effect, i.e., by the metal surface irregularities that facilitated the attack of aggressive ions. The DFT approach showed that the HOMO was located at the benzimidazole part and the LUMO along the whole molecule, except at the molecule ester groups. In addition, the N and S atoms possessed negative charges that are related to molecule steric effects, where the donation of electrons allows the formation of coordinate bonds with the metal, thus being the nucleophilic sites during the adsorption process. The authors concluded that adsorption took place on multiple molecule sites, boosting its stability and IE.

On the other hand, the use of antibiotics as CIs has become very attractive; however, their application is not recommended because of potential consequences such as bacterial resistance, environmental impact and human health damage, as well as their increase in cost and low availability for the population [104]. In this context, Abdel-Hameed et al. [16] studied the use of the drug Co-amoxiclav (CO-AMOX), expired six months before, whose active agents consist of 250 mg of amoxicillin and 125 mg of potassium salt from clavulanic acid. Gravimetric tests with immersion times in the corrosive medium from 1 to 7 h found that CO–AMOX achieved an IE of 90% at 50 ppm and 303 K. In contrast, the acidimetry method reported IEs of 79, 82 and 84% at pH of 1, 2 and 3, respectively, whereas the gasometric tests reported IE values of 76.8, 77.5 and 78.9% under the same conditions.

The authors suggested that the efficiency was due to the synergistic effect of the organic salt, which promoted the adsorption of amoxicillin complexes that blocked the active sites of carbon steel. Like in most of the CI cases for steel, the effect of temperature negatively affected the formation of an inhibiting film consisting of CO–AMOX, thus increasing its desorption and diminishing its stability on the metal surface.

#### Structure-Efficiency Relationship of CIs in H_2_SO_4_ Corrosive Medium

Based on the above analysis, it can be concluded that the corrosion IEs of steel in H_2_SO_4_ were lower than in HCl. Of all the studies mentioned above, only two reported efficiencies greater than 90% in 1 M H_2_SO_4_ at minimum CI concentrations below 26 ppm: compounds based on functionalized pyridine [64] and on pyrimidine-thiols [77]. Like CIs in HCl medium, these compounds contain heteroatoms such as O, N and S, as well as aromatic rings, which have the ability to form covalent/coordinate bonds with the unoccupied d–orbital of Fe and receive free electrons from the metal by means of backdonation [64,77]. In general, the set of CIs analyzed in H_2_SO_4_ medium displayed remarkable structural diversity with compounds featuring thiazoles, thiols, imidazoles and sulfonamide groups, all rich in heteroatoms such as nitrogen, sulfur and oxygen. These functionalities favor the adsorption on metal surfaces through electrostatic interactions, coordinate bonds or mixed mechanisms. Despite the presence of complex structures such as polymers, surfactants and reused drugs, a direct correlation between the molecular size or number of functional groups and IE was not observed. Some simple compounds, such as those derived from thiazole-thiol, have reached IEs comparable to those of more elaborated systems. This fact suggests that the electronic distribution, space orientation of the active groups and compatibility with the acid medium are more determining factors than the structural complexity. Furthermore, the presence of long alkyl chains in cationic surfactants and polymers seems to contribute to the formation of stable protecting films, although this fact does not guarantee by itself a higher IE. The results reinforce the idea that the IE in H_2_SO_4_ depends more on the combination of specific metal–inhibitor interactions and physicochemical properties of the compound than on the number of present functional groups.

### 2.3. Corrosion Inhibition of Carbon Steel in Hydrogen Sulfide

In the oil and gas industry, the environmental and operative conditions are fundamental to establishing the intensity and rate of wet corrosion provoked by hydrogen sulfide (H_2_S). Variables of chemical processes such as temperature, pressure, pH and flow rate are characteristic during the extraction and processing of these resources, notably intensifying this corrosive phenomenon [8]; the right control of these variables is essential for mitigating corrosion promoted by H_2_S. As for more specific properties, the solubility of H_2_S in water increases with temperature and pressure, where deep drilling activities and high–pressure industrial processes (for example, oil refining and natural gas processing) promote conditions that favor the dissolution of H_2_S in water, and then, the formation of hydrosulfuric acid [H_2_S_(ac)_] [105]. This acid works as a concentrated and aggressive corrosive agent in oil pipelines, transforming them into especially vulnerable zones whose continuous exposure triggers susceptibility to internal corrosion and fragility, which could provoke leaks and even catastrophic cracks. Storage tanks are also susceptible to damage and possible content contamination due to H_2_S wet corrosion [24,105]. This type of corrosion notably affects the useful life of equipment, which leads to the necessity of carrying out costly maintenance tasks or even replacements. In addition, the operative efficiency is diminished, which is reflected in a reduction in production and increasing energy consumption [106].

In refining operations, corrosion caused by H_2_S usually starts from sulfur-containing compounds from crude oil, gas wells and gas extracted from oil reservoirs; these compounds include thiophenes, sulfurous alcohols, elemental sulfur, sulfur ethers, disulfides and, of course, H_2_S [24]. It is common to find more complex sulfides such as metal sulfides that can contain multiple sulfur atoms or present elaborate molecular structures. These compounds tend to exhibit a wide variety of chemical properties and reactive behavior, which can represent additional challenges regarding the control of steel corrosion.

The damage by H_2_S_(ac)_ occurs when hydrogen sulfide (H_2_S), a colorless, toxic and flammable gas found naturally in crude oil and natural gas, comes into contact with water. When dissolved, it forms a weak acid that significantly accelerates the corrosion of steel. This mechanism can provoke corrosion in confined spaces (cracks and pitting) and weakening by hydrogen [107]. To summarize, this corrosion mechanism can proceed through four stages as follows [108,109]:

**First stage** (Reaction (16)). Hydrogen sulfide (H_2_S) in aqueous medium (H_2_O) dissociates in positive hydrogen ions (H^+^) and negative bisulfide ions (HS^−^):

(16) $$H_{2}S+H_{2}O↔H^{+}+HS^{-}$$

**Second stage** (Reaction (17)). The bisulfide ion (HS^−^) in aqueous medium produces hydrogen ions (H^+^) and sulfide ions (S^2−^):

(17) $$HS^{-}↔H^{+}+S^{2-}$$

**Third stage** (Reaction (18)). Simultaneously, Fe in the metal oxidizes and produces ferrous ions (Fe^2+^) in anodic zones:

(18) $$Fe\to Fe^{2+}+2e^{-}$$

**Fourth stage** (Reaction (19)). The cathodic species (2H^+^) are reduced to their atomic form (H) and the species produced at stages (16) and (17) react with iron, producing iron sulfide (FeS) as a corrosion product [FeS_x_]. Likewise, hydrogen evolution occurs, producing molecular hydrogen (H_2_):

(19) $$Fe+2H^{+}+S^{2-}\to FeS+H_{2}$$

Hydrosulfuric acid contributes to acidifying the medium. Thus increasing the corrosion rate of steel [110], which becomes more intense in the presence of acids such as H_2_SO_4_ or HCl that are common chemical compounds in industrial wastewater or drilling fluids; it can even be in combination with salty water and other corrosive gases like CO_2_ [111,112]. Specifically, the vulnerability of carbon steels to corrosion in the presence of hydrosulfuric acid is notable and can provoke fast loss of structural integrity because of accelerated corrosion and cracking induced by sulfides [113].

The present review provides Table 3 with a list of CIs with addition at 100 ppm or lower and IE ≥ 90% to protect different types of corrosion steels by H_2_S corrosion.

In the table, ILs derived from pyridinium [114,115,116,117,123,124], quinolinium [114,123], imidazolium [116,117], ammonium [115,116,117,118] and oxazinium [119], as well as some organic compounds derived from amine [118,120], imidazole [121,126], thiazole [125] and polyamides [122] are mainly observed. In general, most CIs of steels in H_2_S and derived compounds include ILs with *Cl*^−^ and *Br*^−^ anions, whereas the cations present higher diversity of structures from simple C_14_ to C_18_ alkyl chains to functionalizations with benzene rings, naphthyl, amine, double bonds, etc., aimed at favoring the adsorption process of the CI on the metal surface.

In this context, ILs work better in H_2_S media due to its unique properties such as thermal and chemical stability, dissolution capacity and high selectivity. These characteristics make them ideal for industrial applications like heavy oil desulfurization and gas capture, improving the efficiencies under extreme conditions [127]. In the first study in Table 3, Iravani et al. [114] analyzed the inhibiting properties of two ILs: dodecyl pyridinium chloride (DDPC) and dodecyl quinolinium chloride (DDQC) for API 5L X65 carbon steel in NACE 1D182 solution. The authors reported IEs of 95.9 and 97.1% at DDPC concentrations of 25 and 50 ppm, respectively. In the case of DDQC, IEs of 94.6 and 96.2% were achieved at the same concentrations. The slight difference found in the results was associated with the participation of the additional benzene ring in the quinolinium group, which affected the cation orientation during the adsorption process of DDQC. Additionally, the temperature was increased (293, 313, 333 and 353 K), observing a negligible decrease in the IE at 100 ppm (ΔIE < 2%), which suggested a chemisorption process due to the ΔG°_ads_ values (−41.8 and −40.9 kJ/mol, respectively). These IEs preserved their value even during WL tests after seven days of immersion. The DFT analysis found that the HOMO and LUMO were distributed in the Cl^−^ anion and pyridinium/quinolinium ring, respectively. On the other hand, the MD studies proposed that the N heteroatoms were key to the molecule ‘anchoring’ on the Fe (110) surface. The authors inferred that the molecule adopted a ‘*decorative*’ orientation, i.e., almost parallel to the Fe surface, while the dodecyl chain was positioned close to the surface.

In contrast, a group of nitrogenated ILs was tested in the corrosion inhibition of Q235 steel along with complementary studies to assess their effectivity as CIs, which was the case of Lu et al. [115] and Zhang et al. [116,117,118]. As for N-cetyl-N,N,N-trimethyl ammonium bromide (CTAB) [115], this IL achieved an IE of 98.4% at 10 ppm protecting Q235 steel immersed in a saturated NaCl solution of H_2_S and CO_2_, whereas imidazoline quaternary ammonium salt (IAS) exhibited an IE > 99% at 30 [116] and 50 ppm [117]. As for N-benzyl pyridinium chloride (BPC), it achieved IEs of 97.1, 99 and 99.7% at concentrations of 10, 20 and 50 ppm [115,116,117], respectively. In the case of CTAB, the obtained IE was higher than those displayed by IAS and BPC, requiring a lower concentration. This result was related to size, long alkyl chain and molecule simplicity, in comparison to the corresponding features of IAS and BPC, which have two aromatic rings that can present steric repulsions between the IL molecules or their partial adsorption attributed to their possible “v” conformation derived from the linking of aromatic rings through a single carbon atom, requiring higher concentration to achieve a high IE. Additionally, CTAB has a Br anion, which has higher ionic radius, electronegativity and polarizability than the anion Cl. A very popular compound was tetradecyl trimethyl ammonium bromide (TTAB), which was evaluated by the previously mentioned authors, achieving an IE > 96% for concentrations starting from 20 ppm at 333 K [115,116,117,118]. Zhang et al. [118] evaluated the synergy between TTAB and octadecylamine (OCT) at different concentrations, OCT:TTAB = 30 ppm, reporting the following results: 5:25 = 99% and 10:20 = 98.7%, where the efficiency was ascribed only to the TTAB results. In contrast, the increase in the OCT proportion modified the IE, diminishing it to 40.1% at 25:5. In the case of these CIs, their IEs were associated with their properties as ILs like high solubility/polarity and thermal/chemical stability [128], as well as with their long alkyl chain, which favored the adsorption process and stability during the residence time on the metal surface. In particular, the adsorption of ammonium-based ILs occurred perpendicularly to the surface, being attracted through the central N atom of the hydrophilic head, whereas the hydrophobic chains remained oriented toward the solution core [129]. On the other hand, ILs with planar conformation like imidazolium and pyridinium tend to be adsorbed parallel to the metal surface, enhancing its adsorption process by means of *π–π* interactions with the steel *d* orbitals [130,131].

The number of ILs employed as CIs to control the corrosion of St3 carbon steel was lower [119]. Regarding conventional CIs, they included organic structures such as acetamide (expired drug) [120], bis-imidazole [121] and a commercial product derived from polyamines with ethylene oxides [122]. Mehdiyeva [119] analyzed oxazinium derivatives, 8-Allyl-3-benzyl-3,4-dihydro-2H-benzo[e][1,3]-oxazin-3-ium bromide (**3e**) and 8-Allyl-3-(4-bromophenyl)-3,4-dihydro-2H-benzo-[e][1,3]oxazin-3-ium bromide (**3f**), in a solution consisting of Stratum waters NACE+ 400 mg/L H_2_S. The author reported IEs of 90 and 92.1% at 50 ppm for the compounds **3e** and **3f**, respectively, which were associated with their water solubility and presence of the anion Br^−^. The slight IE difference was related to the torsion of the molecule caused by the change in the benzyl a 4-bromophenyl group, which by connecting directly both rings, preserved the molecule planar feature. The evaluation of the bactericidal effect of the ILs (IE_3e_ = 87.5% < IE_3f_ = 100%) was also emphasized, which was mainly ascribed to the oxazine group from which these ILs are derived [132].

In this context, Tsygankova et al. [120] analyzed the inhibiting properties of an antiarrhythmic drug: expired lidocaine (2-(diethylamino)-N-(2,6-dimethylphenyl)acetamide). In this study, an IE of 92% at 80 ppm was reported, which was associated with amide, tertiary amine and an aromatic ring, where the two first groups could form coordination bonds, whereas the ring facilitated the planar adsorption of the structure through *π* interactions. In contrast, Berdimurodov et al. [121] evaluated pharmaceutically active glycoluril (GCU), synthesized from low cost products such as urea and glyoxal. An IE of 90.6% at 75 ppm and 303 K was reported as the result of the high number of functional groups and molecule volume. Notwithstanding, the molecule was affected by the temperature, diminishing its IE up to 79.9% at 333 K because of the decreasing E_A_ and endothermal nature of the adsorption process of GCU on the steel surface. The authors proposed that GCU worked through ‘reduction’ by neutralizing the negative regions on the metal surface, adsorbing the protonated N atoms and blocking the active sites with a fine inhibiting film against the attack of corrosive ions such as Cl^−^, HCO_3_^−^, CO_3_^2−^ and HS^−^ available in the medium. In the DFT analysis, a flat and homogenously polarized structure was reported, which promoted strong intermolecular interactions with the metal surface. In contrast, a commercial CI (AMDOR IC-3) was studied by Tsygankova et al. [122], which is a condensation product of tallic acid and polyethylene-polyamines in an alcohol-hydrocarbon solvent and was evaluated in two corrosive media saturated with 400 ppm of H_2_S, M1 or Samotlor oil field solution (g/L: NaCl, 17; CaCl_2_, 0.2; MgCl_2_·6H_2_O, 0.2; NaHCO_3_) and NACE (g/L: NaCl, 5; CH_3_COOH, 0.25). According to what was reported, the CI was more efficient in NACE (91%: 50 mg/L) than in M1 (80%: 50 mg/L), which was probably due the fact that the higher acid NACE medium favored the protonation of the inhibiting compounds. Additionally, the competitive adsorption between the CI and FeS on the metal surface was suggested, which promoted higher localized corrosion; it was concluded that the increase in the CI concentration promoted enhanced surface coverage.

On the other hand, Iravani et al. [123] presented a study of the effect of aromatic rings in ILs derived from quinolinium and pyridinium chlorides: benzyl quinolinium chloride (BQC), naphthyl methyl pyridinium chloride (NMPC) and naphthyl methyl quinolinium chloride (NMQC). From 25 ppm, IEs of 95.9, 96.5 and 97.2% were achieved by BQC, NMPC and NMQC, respectively. The authors associated these results with the electronic density of the NMQC and NMPC aromatic rings (10 *π* electrons), which is higher than in benzyl and pyridinium rings (six *π* electrons), and with the higher area that the naphthyl methyl and quinoline rings can cover. Another aspect to highlight is the low temperature response from 293 to 333 K, presenting ΔIE ~ 2% with seven days of exposure. The DFT analysis showed that the HOMO was located in the pyridinium ring, whereas the LUMO was in naphthyl methyl of NMPC and in pyridinium of BQC and NMQC. The MD study suggested that the adsorption on Fe (110) displayed the following trend: NMQC > NMPC > BQC, confirming the obtained IE by means of PDP and EIS electrochemical techniques. The inhibition mechanism was based on the molecule adsorption by coordination bonds through the naphthyl methyl (NMPC) ring and pyridinium and quinolinium (BQC and NMQC, respectively) rings. Like in other compounds, the anion Cl^−^ in the IL competed against the ion HS^−^ in the medium for occupying the metal positive sites, modifying the charge and allowing the electrostatic attraction of the cation (physisorption). Afterward, the formation of coordination covalent bonds (chemisorption) occurred through *π* bonds between aromatic rings and the empty Fe *d* orbital.

In another work by Berdimurodov et al. [124], the inhibiting properties of a supramolecular IL, Bromide-cucurbit[7]uril (BrCU), were analyzed. The selection of this structure was based on the following points: (1) stability and water solubility, (2) possible cation/anion physical interactions, (3) hydrophobicity of the aliphatic tail and (4) the presence of benzoyl rings, imidazole, carboxyl functional groups and pyridinium cations and bromide anions.

The authors suggested that during the inhibition process, two layers occurred: the first one was formed by CI-Fe complexes and the second one by aliphatic tails oriented toward the solution core, which isolated the metal surface from corrosive ions (Cl^−^, HCO_3_^−^, CO_3_^2−^, HS^−^ and S^2−^), thus limiting the anodic and cathodic reactions. An IE of 92.8% at 75 ppm and 303 K was reported, which fell to 80.7% at 333 K. This result was attributed to the weakening of the electrostatic interaction with Fe at higher temperatures. The DFT analysis ascribed the compound inhibiting properties to the wide planar structure of the IL central part and *p* and *π* electron pairs of the polarized structure. In addition, the HOMO was located around the *Br*^−^ anion, whereas the LUMO was found in the carbon atoms linking two pyridinium rings. Because of the multiple functional groups, a more complex adsorption mechanism was proposed, which featured four adsorption types: (1) physisorption through *Br*^−^ anions and pyridinium N atoms with the medium corrosive ions, (2) chemisorption by the transfer of –COOH *p* electrons to the empty iron *d* orbital, (3) re-chemisorption through the transfer of *π* electrons of pyridinium rings to the empty iron *d* orbital, and (4) backdonation by sharing an electron from the iron *d* orbital with pyridinium rings.

Zhuoke et al. [125] carried out the analysis of bimannich base with thiazole (TZBM) as a potential CI of carbon steel. By WL, IE of 96.4% at 50 ppm and 72 h of exposure was reported, which was associated with the molecule adsorption through lone electron pairs in the N and O heteroatoms. In particular, the possible formation of two adsorption points through six-member rings was emphasized, which reinforced adsorption even at higher temperatures. It should be mentioned that the evaluation at a higher temperature and pressure (453 K + 10 MPa), which significantly increased the system molecular movement, modified the CI attraction and adsorption process because an IE > 90% was kept at concentrations up to 1000 ppm, whereas the inhibitor optimal concentration was 1500 ppm. By SEM analysis, the reported micrographs showed severe surface damage in the absence of TZBM due to the temperature, pressure and corrosive medium, while in the presence of CI, a less damaged surface with the possible deposition of NaCl crystals, was revealed.

#### Structure-Efficiency Relationship of CIs in H_2_S Corrosive Medium

Previous studies have shown that the following compounds presented efficiencies greater than 95% at minimum concentrations of 25 ppm: amines and ILs with cations derived from pyridinium, quinolinium, ammonium and imidazolium, and anions derived from chloride and bromide [114,115,116,118,123]. The presence and number of aromatic rings can modify the orientation of the molecule favorably, thus improving its adsorption. In this case, a parallel distribution of the molecule with respect to the Fe surface is desirable [114,123]. Additionally, longer alkyl chains and simpler molecular structures enhance inhibitory behavior [115,116,118]. Finally, the presence of halides enhances the effectiveness of ILs; Cl^−^ anions compete with HS^−^ ions in the medium to occupy metal positive sites, thereby enabling cation adsorption [114,116,118,123]. However, the Br anion, which has higher ionic radius, electronegativity and polarizability than the Cl^−^ anion, has reached efficiencies above 97% [115]. The CIs analyzed in H_2_S medium presented a marked tendency toward positively charged quaternary structures such as pyridinium, quinolinium, ammonium and imidazolium salts, which in most cases feature long alkyl chains (C_12_–C_18_) that favor the formation of protecting films on carbon steel. These structures allow efficient electrostatic adsorption, especially in the presence of sulfurous species, where polarity and electronic density play a major role. Additionally, nitrogenated and oxygenated heterocyclic compounds such as imidazoles, oxazines, and thiazoles were identified, which provide active sites to the chemical coordination on the metal surface. Despite structural diversity, it is observed that the most effective compounds combine positive charge, molecular flexibility and electron donating groups, which probably allow mixed interactions (physical and chemical) with the metal. The presence of polyamines and ethylene oxides suggests a surface coverage strategy by multiple adsorptions, whereas more complex structures like cucurbiturils could act by encapsulation or blockage of corrosive species. In general, the results indicate that in H_2_S medium, the IE is strongly influenced by the electrostatic adsorption capacity, length of alkyl chains and presence of coordinating heteroatoms and not by the global structural complexity of a compound. The analysis of the performance of CIs in H_2_S reveals that the IE does not depend exclusively on the presence of certain functional groups, but on how they are integrated into structures capable of interacting efficiently with sulfurous species. Quaternary salts with long alkyl chains offer strong electrostatic adsorption, but their performance is improved when combined with heterocycles that provide chemical coordination sites. The synergy among molecular charge, structural flexibility and electronic density allows mixed interaction with the metal, optimizing the surface protection. In addition, the use of polyamines, functionalized surfactants and supramolecular structures such as cucurbiturils suggests that the design of CIs should consider not just the molecular efficiency but also their behavior in dynamic and complex systems. In this context, the H_2_S medium demands formulations that can balance effective adsorption, operative compatibility and stability before corrosive species, which leads the development toward functional compounds strategically assembled beyond the single accumulation of active groups.

## 3. Comparative Analysis of Functional Groups and Heteroatoms in Acid Media

### 3.1. Distribution and Frequency of Functional Groups in CIs

The structural diversity of organic inhibitors evaluated in HCl, H_2_SO_4_ and H_2_S media reveals defined patterns in the recurrence of functional families. These patterns are not random but respond to the physicochemical requirements imposed by every corrosive medium, either governed by protonation, oxidation or interaction processes with sulfurous species. By grouping the compounds in ten main families—such as imidazole derived and triazole-, sulfur-and quaternary-based compounds—it is possible to observe their distribution and establish which functionalities are more compatible with every corrosive acid medium.

**Figure 2:**
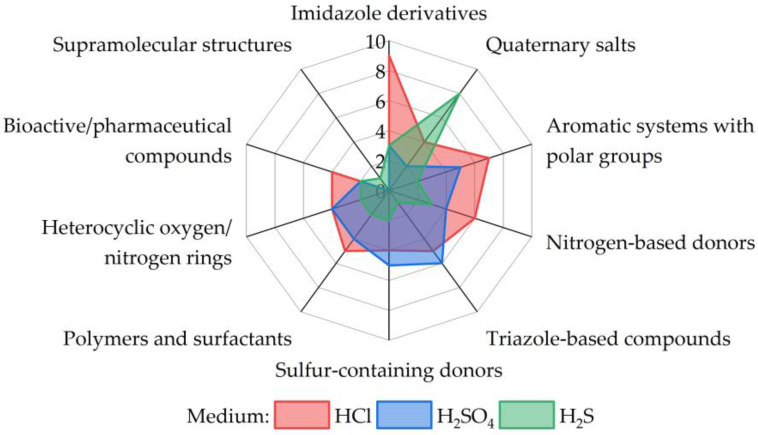
Comparative distribution of families of functional groups in acidic media based on the analyzed articles.

Figure 2 shows the frequency distribution of the functional groups in organic CIs in the three evaluated corrosive acid media. It is known that in HCL, corrosion is strongly influenced by the acidity and protonation capacity; for this reason, in this medium, a high frequency of CIs derived from imidazole, polar aromatic systems and nitrogenated donors is observed, i.e., compounds with free electron pairs from N and O favor the chemical adsorption on carbon steel. The presence of functionalized aromatic rings also allows π–metal interactions, whereas thiols and triazoles provide additional coordination sites. Their IE is due to the localized electronic density and favorable space orientation rather than to the molecular size.

In contrast, corrosion caused by H_2_SO_4_ is more oxidant and highly protonating. The most common CIs in this medium were triazole-based compounds, sulfur-containing donors and oxygen/nitrogen heterocyclic rings, i.e., compounds with multiple heteroatoms. This fact suggests a multisite protection strategy and structural resistance before protonation. Finally, the prevailing CIs in the H_2_S medium were characterized by the presence of quaternary salts and positively charged structures (ILs), thus being electrostatically adsorbed and forming a physical barrier as the main mechanisms.

### 3.2. Distribution and Recurrence of Heteroatoms as Functional Adsorption Centers

The IE of CIs before corrosion in acid media is closely related to the presence and location of heteroatoms in their molecular structures. Nitrogen (N), sulfur (S) and oxygen (O) atoms work as active adsorption centers, being capable of interacting with the metal surface by means of coordinate bonds, electrostatic forces or formation of protecting films.

Based on the foregoing, this section classifies CIs according to the presence of individual heteroatoms (N, S, O) and their combinations: binary ((N–S, N–O, S–O) and ternary (N–S–O), establishing their relative distribution in every acid corrosive medium.

**Figure 3:**
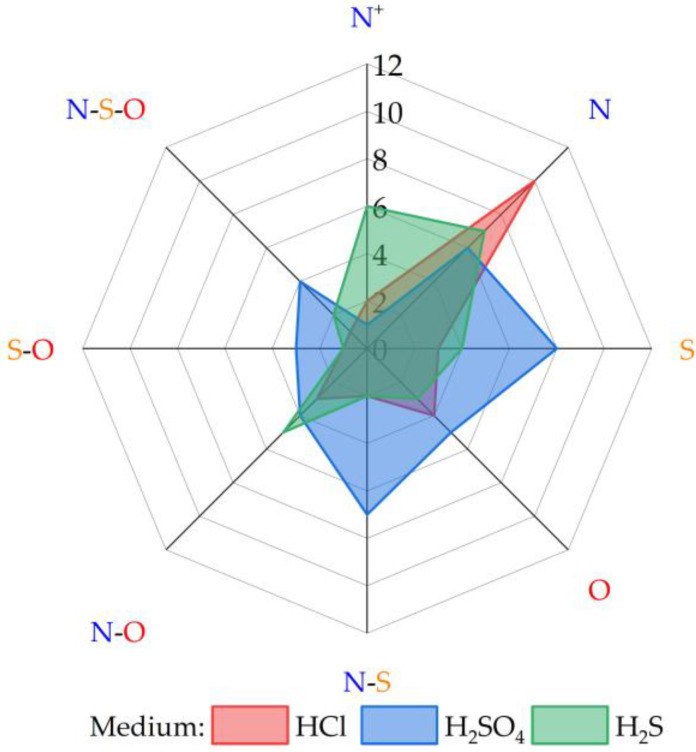
Relative distribution of heteroatom adsorption centers in acidic media based on the analyzed articles.

Figure 3 displays the heteroatom frequencies (N, S, O) and their structural combinations in organic CIs evaluated in HCl, H_2_SO_4_ and H_2_S. It is observed that in HCl, N–based CIs prevail, which helps anticipate a chemical adsorption strategy based on electron donation and surface coordination. In contrast, in H_2_SO_4_, a high recurrence of CIs with sulfur structures (S) and N–S combinations is revealed, which suggests a multisite adsorption mechanism and structural resistance with respect to protonation. As for H_2_S, it is characterized by the prevailing presence of CIs with quaternary ammonium salts (N^+^) and N–O combinations, indicating adsorption and formation of physical barriers/films as more effective inhibition mechanisms for this corrosive medium. It also suggests that N–S–O ternary combinations feature moderate presence, whereas S–O combinations are less frequent and considered more structurally limited.

The functional distribution per heteroatom previously described is a complement of the analysis of functional families because it provides structural criteria for the ‘intentional’ and specific design of CIs depending on the corrosive medium, which allows the prediction of the prevailing interaction between the metal substrate and inhibitor for each corrosive media, mainly oriented toward acid corrosive environments.

### 3.3. Structural Criteria for the Optimized Design of CIs in the Analyzed Media

The distribution patterns of functional groups and heteroatoms identified previously allow us to establish structural criteria for the optimized design of organic CIs specific to each corrosive medium. The recurrence of certain functional groups and combinations of heteroatoms not only reflect chemical compatibility but also efficiency adsorbing the CI, as well as its stability under aggressive conditions in every acid corrosive medium.

In the case of HCl, molecules with nitrogenated centers stand out, which are capable of donating electrons; examples are imidazole-, pyridinium- and quinolinium-derived molecules that present aromatic and planar geometries that favor the direct interaction with the metal surface through coordinate bonds. On the other hand, in H_2_SO_4_, inhibition efficiency is associated with structures that incorporate sulfur atoms and N–S combinations such as aromatic thioureas, oxygenated thiazoles or thiosemicarbazones, which allow adsorption distributed in multiple active sites and offer higher stability with respect to protonation that can cause the medium pH to the organic CI. As for H_2_S, CIs with positively charged groups (N^+^) like imidazolium or quaternary pyridinium salts, along with N–O present in aromatic amides or hydrazides, show a high capacity to establish electrostatic interactions and form protecting films that limit the contact of aggressive species with the metal surface.

The previous examples indicate how the selection or synthesis of CIs can be oriented strategically according to the acid medium, emphasizing characteristics such as electronic density, molecular polarity, heteroatom multifunctionality and conformational flexibility. These criteria allow us to enhance both the efficiency of CIs and their evolution toward more sustainable and compatible formulations with more demanding industrial environments.

## 4. Conclusions

In this review, only organic compounds employed as CIs of carbon steels that achieved IEs above 90% at low concentrations (below 100 ppm) were selected. Most of these compounds contain nitrogen heteroatoms derived from quaternary ammonium or aromatic heterocycles, such as pyridinium, quinolinium and imidazolium. On the other hand, the functionalization of their structures by adding active functional groups, either terminal or attached to their own rings (–NH_2_, –NH–, –N<, –N=N–, =N–, ≡N, –NO_2_, –O–, >C=O, –COOH, –OH, –S–, –SH, =S, >S=O, –SO_2_ and –SO_3_), alkyl chains or halogens stands out as a significant factor for inhibiting metal corrosion in acid media. However, molecular size is not always predictive of high CI efficiencies. As found in this review, there are chemical structures that can reach IEs comparable to those displayed by macro/supramolecules even when they are ‘small’ and with just a few functional groups; this observation emphasizes the fact that the efficient behavior of an inhibitor depends more on compatible interactions with the metal surface than on the structure’s complexity.

The effectiveness of a CI can be significantly affected by an increase in temperature; in contrast, the increase in CI concentration can contribute to the optimal performance of a CI evaluated under more challenging conditions. On the other hand, some CIs could keep and even increase their IE after extended exposure periods, which suggested improved stability despite the time variable. As supporting tools, computational analyses confirmed that rings and/or neutral or protonated heteroatoms are regions through which a given CI can be adsorbed by physisorption and/or chemisorption via *π* electrons or lone electron pairs. 

## Figures and Tables

**Table 1:** Corrosion inhibitors for steel in HCl with optimum efficiencies (IE ≥ 90%).

Abbreviation Name	Chemical Structure	Metal/ HCl Conc.	CI_C_ IE (%)	Ref.
**DHAA** Dehydroabietylamine	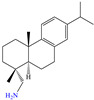	Mild steel/1.0 M HCl	25 ppm: 91.0	[28]
**MF3** 1,5-dimethyl-1H-pyrazolo[3,4-d]pyrimidin-4(5H)-one	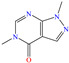	Mild steel/1.0 M HCl	1 × 10^−4^ M (~16.4 ppm): 90.5	[29]
**3-OYA** 3-(1,3-oxazol-5-yl)aniline	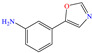	Mild steel/1.0 M HCl	0.4 mM (~64 ppm): 92.8	[30]
**DDD** 2,2^1^ disulfane-diyl-dianiline	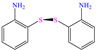	Mild steel/0.5 M HCl	40 ppm: 96	[31]
**PTU** N-phenylthiourea	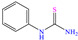	Mild steel/1.0 M HCl	1 × 10^−4^ M (~15.2 ppm): 90.5	[32]
**MTSC** 1-mesitylethanone thiosemicarbazone	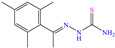	Mild steel/1.0 M HCl	0.3 mM (~70.6 ppm): 92.3	[18]
**T3** 1-octyl-2-(octylthio)-1H-benzimidazole	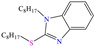	Mild steel/1.0 M HCl	1 × 10^−4^ M (~37.4 ppm): 90.4	[33]
**Q-NO2** 3,3-dimethyl-12-(4-nitrophenyl)-3,4,5,12-tetrahydrobenzo[4,5]imidazo[2,1-b]quinazolin-1(2 H)-one	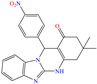	Mild steel/1.0 M HCl	1 × 10^−4^ M (~38.8 ppm): 93.5	[34]
**Q-OMe** 12-(4-methoxyphenyl)-3,3-dimethyl-3,4,5,12-tetrahydrobenzo[4,5]imidazo[2,1-b] quinazolin-1(2H)-one	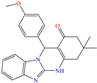		1 × 10^−6^ M (~0.3 ppm): 91.5	
**COMI** 2-(3-chloro-2-(4-hydroxy-3,5-dimethoxyphenyl)-4-oxoazetidin-1-yl)-1-methyl-1H-imidazole-4(5H)-one	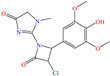	Mild steel/1.0 M HCl	20 ppm + 333 K: 95.5	[21]
**EIDTC2ClBuSn** Bis(N-ethyl-isopropyl dithiocarbamate)chlorido butyltin (IV)	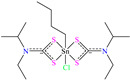	Mild steel/1.0 M HCl	0.001 mM (~0.5 ppm): 96.9	[35]
**ZM6** 14-(4-nitrophenyl)-14H-dibenzo[a,j]xanthene	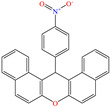	Mild steel/1.0 M HCl	1 × 10^−4^ M (~40.3 ppm): 91.3	[36]
**Tetracycline** (4S,4aS,5aS,12aS)-4-(Dimethylamino)-1,4,4a,5,5a,6,11,12a-octahydro-3,6,10,12,12a-pentahydroxy-6-methyl-1,11-dioxo-2-naphthacenecarboxamide	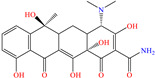	Mild steel/1.0 M HCl	2 × 10^−6^ M (~0.8 ppm) + 273.15 K: 91.3	[37]
**KETO** Ketoconazole	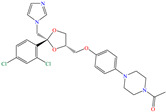	Mild steel/1.0 M HCl	50 ppm: 93.4	[38]
**EA** Expired atorvastatin	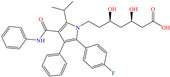	Mild steel/1.0 M HCl	50 ppm: 94.4	[39]
**HMIBr** 1-Hexadecyl-3-methylimidazolium bromide	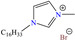	Mild steel/1.0 M HCl	2 × 10^−4^ M (~77.5 ppm): 94.5	[7]
2-decyl phosphate-1-(N,N-dimethyl,N-tetradecylammonium) ethane	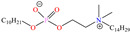	Mild steel/0.5 M HCl	10 ppm + 298 K: 93	[40]
**Copolymer POPS** Polydimethylsiloxane (PDMS)+ polyoxyalkylene (POA)	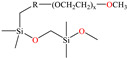	Mild steel/2.0 M HCl	5 × 10^−6^ M (~85 ppm): 93.8	[41]
**DETA-GO** Diethylenetriamine functionalized graphene oxide	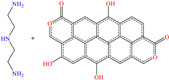	Mild steel/1.0 M HCl	25 ppm: 92.7	[4]
**PGA** Poly-γ-glutamic acid	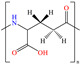	Mild steel/1.0 M HCl	50 ppm + 293 K: 90.2	[42]
**Gelatin** Polypeptide chain	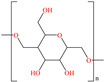	Mild steel/1.0 M HCl	25 ppm + 303 K: 90.5	[43]
**NAC** N-acetyl cysteine	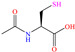	Mild steel/15% HCl	1.0 mM (~163.2 ppm) + 303 K: 93.7	[26]
		X80 steel/15% HCl	0.5 mM (~81.6 ppm) + 303 K: 91.6	
**BDMA** 1-(benzo[d]thiazol-2-yl)-3-chloro-4-(3,5-dichlorophenyl)-4-methylazetidin-2-one	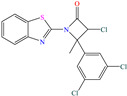	N80 steel/15% HCl	40 ppm: 92.3	[6]
**BDMT** 3-(benzo[d]thiazol-2-yl)-2-(3,5-dichlorophenyl)-2,5-dimethylthiazolidin-4-one	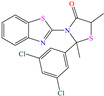		30 ppm: 90.3	
**PzMBP** 2-(1-((piperazine-1-yl)methyl)-1H–benzo[d]imidazol-2-yl)phenol	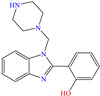	N80 steel/15% HCl	32.3 × 10^−5^ M (~ 99.6 ppm) + 303 K: 90.8	[11]
**IMSQ** Imidazoline phenyl thiourea iodized n-butane quaternary ammonium salt	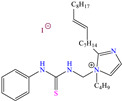	N80 steel/15% HCl	0.03 mM (~20 ppm): 96.7	[13]
**OMID** (Z)-2-(2-(heptadec-8-en-1-yl)-4,5-dihydro-1H-imidazol-1-yl)ethan–1-amine	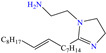	Carbon steel/1.0 M HCl	0.01 mM (~3.5 ppm): ~96	[44]
**Terazosin** [4-(4-amino-6,7-dimethoxyquinazolin-2-yl)piperazin-1-yl]–(tetrahydrofuran-2-yl)methanone	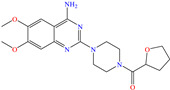	1018 carbon steel/1.0 M HCl	50 ppm: 91.4	[45]
**Br-BBD** 2-(p-bromobenzylthio)-1H-benzimidazole	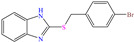	Q235 carbon steel/1.0 M HCl	40 ppm: 90.5	[5]
**LCFH** Long chain fatty hydrazides	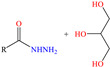	Q235 carbon steel/1.0 M HCl	10 ppm: 92.73	[46]
**SLS** Sodium lignosulfonate	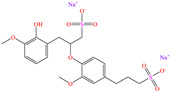	Q235 carbon steel/1.0 M HCl	10 ppm: 96.2	[47]
**DMC-NL** Modified nano-lignin corrosion inhibitor	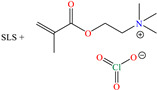	Q235 carbon steel/1.0 M HCl	8 ppm: 92.5	[48]
**UPy-D400-PEGDA** Ureidopyrimidinone + Poly(amino ester)	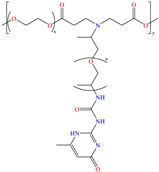	Q235 carbon steel/1.0 M HCl	50 ppm: 91.4	[12]
**TBDB** N1, N1, N4, N4-tetramethyl-N1,N4-bis(2-(hexadecanoyloxy)ethyl)butane-1,4-diammonium bromide	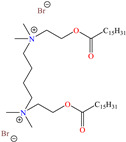	Q235 carbon steel/1.0 M HCl	20 ppm: 94.6	[1]
**NDI-N-Br** 3,3′-(1,3,6,8-tetraoxo-1,3,6,8-tetrahydrobenzo[lmn][3,8]phenanthroline-2,7-diyl)bis(N-ethyl-N,N-Dimethyl-1-propylamine)bromide	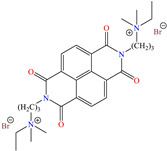	Q235 carbon steel/1.0 M HCl	50 ppm: 90.3	[49]
**PGEPPP** Pentaglycidyl ether pentaphenoxyphosphorus	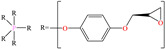	C38 steel/1.0 M HCl	0.1 mM (~85.6 ppm): 93.2	[50]
**T1** N,N′-[2,2′-thiocarbonylbis(hydrazine-2,1-diyl)bis(thioxomethylene)]dibenzamide	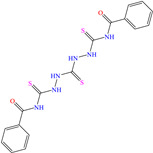	Carbon steel/1.0 M HCl	1 × 10^−4^ M (~43.2 ppm): 92.7	[51]
**T2** N,N′-[2,2′-thiocarbonylbis(hydrazine-2,1-diyl)bis(thioxomethylene)]bis(4-methoxybenzamide)	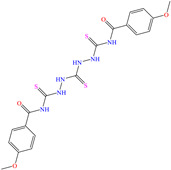		5 × 10^−5^ M (~24.6 ppm): 95.9	
**3c** 1-[3-oxo-3-[(4-oxo-2-pheylquinazolin-3(4H)-yl)amino]nonil]pyridinium bromides	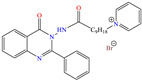	Carbon steel/1.5 M HCl	10 ppm: 93.6	[52]
**Fluconazole** 2-(2,4-difluorophenyl)- 1,3-bis(1*H*-1,2,4-triazol-1-yl) propan-2-ol	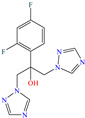	Steel/1.0 M HCl	50 ppm + 296 K: 92.3	[53]

CIc = Corrosion inhibitor concentration.

**Table 2:** Corrosion inhibitors of steel corrosion in H_2_SO_4_ with optimal efficiencies (IE ≥ 90%).

Abbreviation Chemical Name	Chemical Structure	Metal/ H_2_SO_4_ Conc.	CI_C_: IE (%)	Ref.
**Polymer AMTP** Poly[(2,6-dicarbonylpyrdine) (2,5-dihydrazinyl-1,3,4-thiadiazole)]	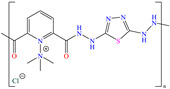	C1018-steel/ 1.0 M	100 ppm: 90.3	[67]
**AS** 4-((4-hydroxy-3-((pyridine-2-ylimino)methyl)phenyl)diazenyl)benzonitrile	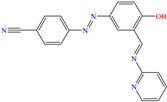	Carbon steel/ 1.0 M	0.08 mM (~26.2 ppm): 91.32	[64]
**C3** 3-(3-Methyl-1-phenyl-1H-pyrazolo[3,4-d]pyrimidin-4-yloxy)-propionic acid hydrazide	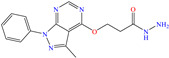	Carbon steel/ 1.0 M	50 ppm: 91.1	[75]
**ERT** N2, N4, N6-tris(2-(oxiran-2-yl methoxy)ethyl)-N2,N4,N6-tris(oxiran-2-yl methyl)-2.4.6-triamine-1,3,5-triazine	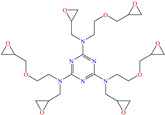	Carbon steel/ 0.5 M	1 × 10^−5^ M (~5.9 ppm): 90.7	[78]
**MBTTA** Methyl 2-(benzamido)-2-(4-p-tolyl-1H-1,2,3-triazol-1-yl)acetate	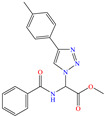	Mild steel/ 1.0 M	0.25 mM (~87.5 ppm): 90.13	[79]
**Urispas** 2-(1-piperidyl)ethyl 3-methyl-4-oxo-2-phenylchromene-8carboxylate	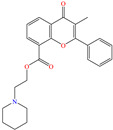	Mild steel/ 1.0 M	50 ppm: 94.03	[81]
**PTM** 4-(4-Methylphenyl)-6-phenyl-3,4-dihydropyrimidine- 2(1H)-thione	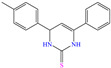	Mild steel/ 1.0 M	0.05 mM (~14 ppm): 91.8	[77]
**PTMO** 4-(4-Methoxyphenyl)-6-phenyl-3,4-dihydropyrimidine- 2(1H)-thione	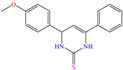		0.1 mM (~29.6 ppm): 96.3	
**TDB** 1,16-(5,12-dihydroxy-7,10-dioxa-3,14-diazahexadecane)di-p-benzenesulfonamide	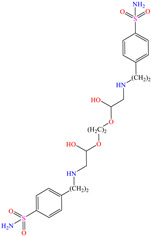	Mild steel/ 0.5 M	50 ppm: 94.6	[17]
**DBIB** 2,2′-dibenzimidazolyl butane	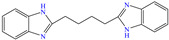	Mild steel/ 0.5 M	50 ppm: 92	[82]
**AMTT** 4-amino-5-methyl-4H-1,2,4-triazole-3-thiol	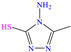	Mild steel/ 0.5 M	25 × 10^−6^ M (~3.3 ppm): 94	[15]
**PAP** 1-phenyl-3-amino-5-pyrazolone	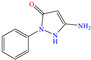	Mild steel/ 0.5 M	0.05 mM (~8.7 ppm): 95.1	[83]
**Triton X-100** Polyethylene glycol octylphenol ester	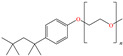	Carbon steel OL 37/ 0.5 M	50 ppm: 94	[84]
**Triton A-20** [4-(2,4,4-trimethyl Pentan-2-yl)phenol + formaldehyde + oxirane]	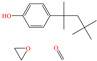		50 ppm: 91	
**Tween 60** [Polyoxyethylene(20)sorbitan monostearate]	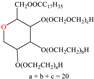	Carbon steel OL 37/ 0.5 M	50 ppm: 92	[85]
**Tween 80** [Polyoxyethylene(20)sorbitan monooleate]	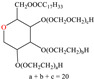		50 ppm: 94	
**SSD** 1-Dodecane sulfonate sodium	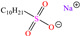	Carbon steel OL 37/ 0.5 M	50 ppm: 93	[86]
**MTATT** 5-methyl-4-[(thiophene-2-yl-methylidene)amino]-4H-1,2,4-triazole-thiol	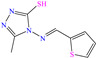	Maraging steel of 18% Ni M250/ 0.2 M HCl + 0.1 M H_2_SO_4_	50 ppm + 318 K: 97.67	[80]
**DBAMTT** 4-{[4-(dimethyl-amino)benzylidene] amino}-5-methyl-4H-1,2,4-triazole-3-thiol	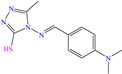		75 ppm + 318 K: 96.99	
**MTBU-I** 1-((1-(4-iodobenzyl)-1H-1,2,3-triazol-4-yl)methyl)pyrimidine-2,4-(1H,3H)-dione	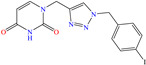	API 5L X52 steel/ 1.0 M	50 ppm: 93.6	[76]
**MTBT-Br** 1-((1-(4-bromobenzyl)-1H–1,2,3-triazol-4-yl)methyl)-5-methylpyrimidine-2,4-(1H,3H)-dione	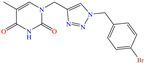		50 ppm: 95.4	
**(EMIM)^+^(SCN)^−^** 1-Ethyl 3-methylimidazolium thiocyanate	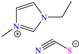	API 5L X52/ 0.5 M	75 ppm: 90	[87]
			10 ppm + 328 K: 93.1	[88]
**GS** 3,3′-(decane-1,10-diyl)bis (1-hexadecyl-1H-imidazol-3-ium)dibromide	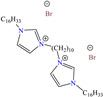	Carbon steel/ 0.5 M	50 ppm: 90.5	[63]
**Cetrimide** Cetyltrimethylammonium bromide	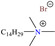	Carbon steel/ 0.5 M	0.2 mM (~72.9 ppm): 92.25	[89]
**DMEC_16_N-ES** N,N-dimethyl-N-hexadecyl-N-ethyl-ammonium ethyl sulfate	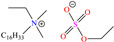	API 5L X60 steel/ 1.0 M	75 ppm: 91	[90]
**OP** Omeprazole	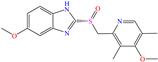	Q235 steel/ 0.1 M	5 × 10^−5^ M (~17.3 ppm): 96.31	[91]
**CO-AMOX** Amoxicillin + Potassium salt of clavulanic acid	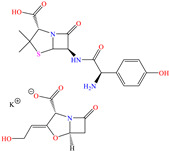	Carbon steel/ 1.0 M	WL + 7 h: 50 ppm: 90	[16]

CIc = Corrosion inhibitor concentration.

**Table 3:** Corrosion inhibitors for steel in H_2_S with optimum efficiencies (IE ≥ 90%).

Abbreviation Chemical Name	Chemical Structure	Metal/ Medium	CI_C_ IE (%)	Ref.
**DDPC** Dodecyl pyridinium chloride	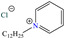	API 5L X65 carbon Steel/NACE 1D182 solution	25 ppm: 95.9	[114]
**DDQC** Dodecyl quinolinium chloride	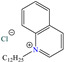		25 ppm: 94.6	
**CTAB** N-cetyl-N,N,N-trimethyl ammonium bromide	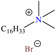	Q235 steel/H_2_S/CO_2_/NaCl solutions	10 ppm: 98.4	[115]
**IAS** Imidazoline quaternary ammonium salt	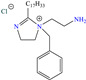	Q235 steel/3.5 wt.% NaCl + CO_2_/H_2_S (333 K)	30 ppm: 99.2	[116]
			50 ppm: 99.5	[117]
**BPC** N-benzyl pyridinium chloride	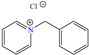	Q235 steel/H_2_S/CO_2_/NaCl solutions	10 ppm: 97.1	[115]
		Q235 steel/3.5 wt.% NaCl + CO_2_/H_2_S (333 K)	20 ppm: 99	[116]
			50 ppm: 99.7	[117]
**TTAB** Tetradecyl trimethyl ammonium bromide	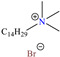	Q235 steel/H_2_S/CO_2_/NaCl solutions	20 ppm: 96.7	[115]
		Q235 steel/3.5 wt.% NaCl + CO_2_/H_2_S (333 K)	30 ppm: 98.9	[116]
		Q235 steel/3.5 wt.% NaCl + CO_2_/H_2_S (333 K)	50 ppm: 99.6	[117]
		Q235 steel/20 mL/min H_2_S + 20 mL/min CO_2_ + 3.5 wt.% NaCl solution (333 K)	30 ppm: 97.6	[118]
**OCT** Octadecylamine	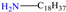	Q235 steel/20 mL/min H_2_S + 20 mL/min CO_2_ + 3.5 wt.% NaCl solution (333 K)	5 ppm OCT + 25 ppm TTAB: 99.0	[118]
**3e** 8-Allyl-3-benzyl-3,4-dihydro-2H-benzo[e][1,3]-oxazin-3-ium bromide	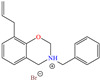	Carbon steel St3/H_2_S saturated water-salt-hydrocarbon medium	50 ppm: 90.0	[119]
**3f** 8-Allyl-3-(4-bromophenyl)-3,4-dihydro-2H-benzo-[e][1,3]oxazin-3-ium bromide	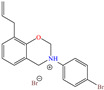		50 ppm: 92.1	
**Expired Lidocaine** 2-(diethylamino)-N-(2,6-dimethylphenyl)acetamide	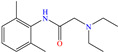	Carbon steel St3/Stratum waters NACE + 400 mg/L H_2_S solution	80 ppm: 92	[120]
**GCU** (3ar,6ar)-3a,6a-bis(4-((3,5-bis(2-(3-(tert-butyl)phenyl)propan-2-yl)phenyl)diazenyl)phenyl)tetrahydroimidazo[4,5-d]imidazole-2,5(1H,3H)-dione	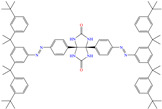	Carbon steel St3/1 M NaCl saturated with CO_2_ + H_2_S	75 ppm + 303 K: 90.1	[121]
**AMDOR IC-3** Fatty tallow polyamines + ethylene oxides in an alcohol solvent	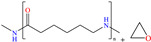	Carbon steel St3/NACE saturated with H_2_S (400 mg/L)	50 ppm: 91	[122]
**BQC** Benzyl quinolinium chloride	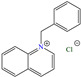	Carbon steel/NACE 1D182 solution	25 ppm: 95.9	[123]
**NMPC** Naphthyl methyl pyridinium chloride	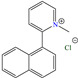		25 ppm: 96.5	
**NMQC** Naphthyl methyl quinolinium chloride	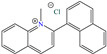		25 ppm: 97.2	
**BrCU** Bromide-cucurbit[7]uril	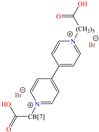 Where 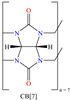	Carbon steel/1 M NaCl saturated with CO_2_ + H_2_S	75 ppm: 92.8	[124]
**TZBM** Bimannich-based containing a thiazole ring	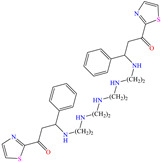	Carbon steel/Gas-liquid environment with Cl^−^ + H_2_S + CO_2_ + 453 K	50 ppm: 96.4	[125]

CIc = Corrosion inhibitor concentration.

## Data Availability

No new data were created or analyzed in this study. Data sharing is not applicable.
